# Initial psychometric evaluation of the Lipedema Quality of Life questionnaire in females with lipedema in Norway

**DOI:** 10.3389/fgwh.2026.1917274

**Published:** 2026-10-09

**Authors:** Julianne Lundanes, John Roger Andersen, Jutta Dierkes, Klaus Schrader, Siren Nymo

**Affiliations:** 1 Nord-Trøndelag Hospital Trust, Clinic of Surgery, Namsos Hospital, Namsos, Norway; 2 Department of Health and Caring Sciences, Western Norway University of Applied Sciences, Førde, Norway; 3 Department of Research and Innovation, Førde Hospital Trust, Førde, Norway; 4 Centre for Nutrition, Department of Clinical Medicine, University of Bergen, Bergen, Norway; 5 Medical Practice for Vascular Diseases, Hof, Germany; 6 Department of Public Health and Nursing, Faculty of Medicine and Health Sciences, Norwegian University of Science and Technology (NTNU), Trondheim, Norway

**Keywords:** function, prom, psychometric, reliability, womens health

## Abstract

**Background:**

There are no validated Norwegian disease-specific patient-reported outcome measures (PROMs) assessing quality of life (QoL) in females with lipedema, limiting evaluation of treatment effect in clinical practice and research.

**Objective:**

To translate and validate a Norwegian translation of the LIPQOL questionnaire.

**Methods:**

A cross-sectional validation study was performed in 432 females with lipedema in Norway. Descriptive statistics and floor and ceiling effects were assessed. The psychometric properties of the questionnaire were assessed using confirmatory factor analysis (CFA). The overall goodness of fit was evaluated using the root mean square error of approximation (RMSEA), the comparative fit index (CFI) and the Tucker–Lewis index (TLI). Internal consistency was assessed using composite reliability from the CFA. Associations between LIPQOL subscales and RAND-12 physical component score (PCS) and mental component score (MCS) were examined using Spearman correlations.

**Results:**

The LIPQOL questionnaire comprises four subscales, Function, Body image, Symptoms, and Feelings. No subscales was affected by floor or ceiling effects. The unmodified CFA shows CFI = 0.929, TLI = 0.922, standardized root mean square residual (SRMR) = 0.073, and RMSEA = 0.079 (90% CI: 0.073 to 0.084). All composite reliability were considered reasonable (ranged from 0.890 to 0.916). Modification indices were evaluated, and two modifications were performed between items 5 and 8, and between items 17 and 18. With modification indices, the overall structure validity showed reasonable, but not unequivocally support for the four factor structure with CFI = 0.944, and TLI = 0.938, SRMR = 0.068, and RMSEA = 0.070 (90% CI: 0.064 to 0.075). Correlation between RAND-12 and LIPQOL subscales were large. Function, Symptoms, and overall quality of life show large correlation with PCS (Spearman's rho = −0.682, Spearman's rho = −0.550, and Spearman's rho = 0.554, respectively) and the subscales feelings and overall quality of life shows large correlation with MCS from RAND-12 (Spearman's rho = −0.685 and 0.608, respectively).

**Conclusion:**

Initial findings suggest that the Norwegian version of the LIPQOL is a reasonably valid instrument for assessing quality of life in females with lipedema, although the findings do not provide unequivocal support for all aspects of its measurement properties.

## 1 Introduction

Lipedema is a disease that predominantly affects females. It is often misdiagnosed as obesity and is characterized by a painful disproportionate accumulation of adipose tissue in the legs, and sometimes in the arms as well. Females with lipedema report reduced quality of life (QoL) compared to the general population, especially in domains such as physical functioning, pain, social functioning, emotional well-being, and energy (1).

Consequently, assessing QoL in this patient group requires appropriate measurement tools. Research on QoL in different patient groups requires the use of both generic and disease-specific questionnaires, often referred to as patient-reported outcome measures (PROMs) (2). Generic tools are important to enable comparison across different patient groups and with the general population (3). Disease-specific questionnaires, on the other hand, are crucial for capturing changes in symptoms and function within domains that are particularly relevant to the patient group in question (3).

Studies mapping QoL in females with lipedema have previously used the Lymphedema quality of life (LYMQOL) questionnaire (4, 5), which is originally developed for patients with lymphedema, or general tools such as RAND-36/SF-36 or WHOQOL-BREF (1). Moreover, the LYMQOL questionnaire has also been validated for females with lipedema in Germany (6), although it includes items irrelevant for lipedema. Lymphedema and lipedema are often confused, although the pathophysiology differs substantially (7, 8),. Even though lipedema-specific PROMs addressing symptom burden have been developed (9), there is currently no disease-specific validated Norwegian QoL questionnaire specifically for females with lipedema. Therefore, the aim of this study was to translate and validate a disease-specific QoL questionnaire, Lipedema Quality of Life questionnaire (LIPQOL), in a Norwegian population. The hypothesis is that the LIPQOL shows reasonable model fit in CFA analysis.

## 2 Methods

### 2.1 Study design

The translation and psychometric evaluation of the Norwegian LIPQOL were guided by contemporary recommendations for PROM development and evaluation, including the COSMIN methodological framework, with adaptations to the procedures where appropriate. A detailed description of the procedures and their relation to the COSMIN recommendations is provided in supplementary Table 1. The main methodological steps are summarized below.

**Table 1:** Participant characteristics from self-report (*n* = 432).

Variables	Mean (SD)
Weight, kg	88.7 (19.6)
Height, cm	166.8 (5.6)
BMI, kg/m^2^	31.9 (6.8)
	N (%)
Age	
18–29	15 (4%)
30–45	99 (23%)
46–64	260 (60%)
> 65	55 (13%)
Missing	3 (1%)
Menstruation status	
Yes, regular	97 (23%)
Yes, but irregular	48 (11%)
No	255 (59%)
Not sure	24 (6%)
Do not wish to answer	6 (1%)
Missing	2 (1%)
Education	
Upper secondary school/vocational education	162 (38%)
College/University	268 (62%)
Missing	2 (1%)
Employment/full time student	
Yes	274 (63%)
No	156 (36%)
Missing	2 (1%)
Cohabitation status	
Living alone	74 (17%)
Living with someone	357 (83%)
Missing	1 (0%)
Perceived home practicality	
Very practical and easy to maintain	113 (26%)
Quite practical and easy to maintain	163 (38%)
Neither nor	94 (22%)
Somewhat impractical and difficult to maintain	47 (11%)
Very impractical and difficult to maintain	14 (3%)
Missing	1 (0%)
Exercise frequency	
Never	12 (3%)
< 1 time per week	43 (10%)
1 time per week	73 (17%)
2–3 times per week	210 (49%)
Every day	93 (22%)
Missing	1 (0%)
Usual exercise intensity	
No sweat	161 (37%)
Sweat	232 (54%)
Exhausted	15 (4%)
Missing	24 (6%)
Exercise duration per session	
< 15 min	16 (4%)
15–29 min	91 (21%)
30–60 min	250 (58%)
> 60 min	53 (12%)
Missing	22 (5%)

Data presented as *n* (%).

A cross-sectional study was performed among females with lipedema in Norway. Clinical variables as age group, menstruation and menopause status, education level, employment status, cohabitation status, home experience, exercise frequency, exercise intensity, and exercise duration were collected. Body weight and height were self-reported. They were also asked if they have received an official lipedema diagnosis, the use of different treatment options, and the time from symptom debut to diagnosis.

### 2.2 PROM instruments

The German version of the LIPQOL was previously developed with LYMQOL as inspiration (10), but a full validation article has not been published. Our initial intention was to develop a new disease-specific quality-of-life questionnaire for people with lipedema in Norway. As part of this preparatory work, the research team discussed with clinicians and patient representatives which aspects of living with lipedema should be represented in such an instrument. During this process, we became aware of the recently developed German LIPQOL and therefore critically examined its content against the aspects identified as important, as well as current knowledge of the condition, its clinical manifestations, and patient experiences. We found that the LIPQOL corresponded well with the content considered relevant for a disease-specific quality-of-life measure. Rather than developing a new and potentially overlapping instrument, we therefore decided to translate the LIPQOL and proceed with psychometric evaluation of a Norwegian version.

The LIPQOL was received with permission from the original developer, and then was translated from German into Norwegian by three Norwegian researchers with basic German skills, assisted by artificial intelligence–based translation tools, including ChatGPT-5 and Microsoft Copilot. This translation process was informed by recent recommendations concerning the use of AI-assisted methods in questionnaire translation (11). The researchers worked collaboratively to ensure correspondence in wording, meaning, and conceptual content between the German and Norwegian versions. The translation was subsequently reviewed by a nutrition researcher who is a native German speaker with strong Norwegian language skills. Finally, two patient representatives reviewed the Norwegian version, considering the wording, meaning, relevance, and comprehensibility of the items from a patient perspective and providing input to the final version.

One modification was made to the response structure of the original questionnaire. The German version includes a “Not applicable” option, indicating that respondents do not need to answer an item when it is not relevant to their situation. For the Norwegian validation study, this explicit response option was removed to encourage respondents to provide responses whenever an item was applicable and thereby maximize the amount of available data. In considering this modification, we judged that the large majority of items were relevant and could be meaningfully answered by all patients in the target population. We identified two items for which a genuinely non-applicable situation could reasonably arise: the item concerning compression garments, for respondents who did not use them, and the item concerning the impact of lipedema on a relationship, for respondents without a partner. Respondents were not required to answer all items and could therefore leave these or other individual items unanswered. Unanswered items were treated as missing and excluded from the calculation of the domain score. This corresponds to the scoring consequence of selecting “Not applicable” in the original German version, where the item is likewise excluded from the domain score.

The Norwegian versions of the LIPQOL and the RAND-12 questionnaires were distributed via the online platform Questback (Oslo, Norway) to females with lipedema. The survey was distributed via the Norwegian Lymphedema and Lipedema Association's (NLLF) email registry, which included approximately 2,400 individuals with either lipedema or lymphedema. The first question in the Questback survey asked respondents to indicate whether they had lipedema, lymphedema, both conditions, or neither. Those reporting only lymphedema or neither condition were deemed ineligible and were therefore not routed to the main questionnaire. This is the first full psychometric assessment of both the instrument and translated version. The LIPQOL consists of 3 single items (Item 1,2 and 26), and 4 subscales; Function (Items 3a- 3f, 4 and 5), Body image (items 6–13), Symptoms (items 14–19), and Feelings (items 20–25). Each subscale score is calculated as the mean of its completed items. More than 50% of the items within a domain had to be completed for a domain score to be calculated.

RAND-12 was assessed for convergent validity testing, and the physical component score (PCS) and mental component score (MCS) was applied. The RAND-12 is a widely used and freely available measure of generic health-related QoL and is strongly correlated with the longer version; RAND-36. RAND-12 shows satisfactory validity (12).

### 2.3 Statistical analysis

Statistical analysis was performed using STATA (StataCorp LLC version 18.5, College Station, Texas, USA). Clinical and sociodemographic data are described as number and percentages or means and standard deviation (SD). Floor and ceiling effects were calculated as the percentage of participants responding to the lowest or highest possible scores. If this number is >15%, this effect seem to be present (13). Construct validity was assessed using confirmatory factor analysis (CFA) for ordinal data with weighted least squares and mean and variances (WLSMV) using JASP version 0.95.4 (JASP, 2024). Factor loadings <0.4 were considered poor. The overall goodness of fit was assessed using the root mean square error of approximation (RMSEA), standardized root mean square residual (SRMR), the comparative fit index (CFI), and the Tucker–Lewis index (TLI). Model fit was indicated by RMSEA close to or below 0.06, SRMR close to or below 0.08, and by CFI as well as TLI close to or above 0.95 (14). The CFA was performed with *post-hoc* modification indices adjustments and without. Adjustments were selected based on an overall assessment of the magnitude of the modification indices and whether the suggested modifications were theoretically justifiable. Missing data were handled using listwise deletion for the CFA Factor loadings from the CFA were used to calculate composite reliability (CR) values for the subscales, with scores ≥0.70 being considered acceptable (15). The following formula was used to calculate CR: $\frac{{\left(\sum standardized\;factor\;laodings\right)}^{2}}{{\left(\sum standardized\;factor\;loadings\right)}^{2}+\sum error\;variances}$.Convergent validity was investigated by assessing correlation between the subscales from LIPQOL and PCS and MCS from RAND-12. We hypothesized that better LIPQOL scores would be positively correlated with better RAND-12 PCS and MCS scores. Correlations was interpreted as small if r = 0.10, medium if r = 0.3 and large if = 0.5 (16).

### 2.4 Sensitivity analysis for missing data

The primary CFA was estimated using WLSMV for the ordinal indicators and was based on complete cases, as the CFA procedure for ordinal data in JASP does not handle missing data. Because missing responses were mainly in items that could conditionally not apply, we conducted an additional sensitivity analysis to assess whether excluding participants with incomplete data affected the results. We re-estimated the same four-factor model using maximum likelihood (ML) estimation with and without full information maximum likelihood (FIML), which allows participants with partially missing item data to be included in the analysis. Although ML estimation is not the method of choice for ordinal indicators and may produce biased estimates when ordinal data are treated as continuous, it was not used as an alternative to the main WLSMV analysis. Instead, we carried out the FIML analysis solely as a sensitivity analysis to examine the possible impact of missing data. We compared model fit and parameter estimates descriptively across the two analyses.

### 2.5 Ethics

Regional Ethics Committee (REC) confirmed that no ethical approval was required, as only anonymous data were collected. No IP-addresses were collected during questionnaire completion. There was no direct contact between the researchers and the participants during data collection. The questionnaire was distributed electronically through Questback, accompanied by a written invitation to participate and information about the study. Before accessing the questionnaire, participants were required to click “OK” on a statement confirming that “by proceeding, you consent to participate.” Submission of a completed questionnaire was therefore considered to constitute informed consent, in accordance with the Declaration of Helsinki.

## 3 Results

A total of 494 participants responded (response rate 21%), of whom 62 were excluded because they did not have lipedema, leaving 432 participants with lipedema to be included in the study. Characteristics of the participants are presented in Table 1. Most participants were aged 46 to 64 years (60%) Lipedema diagnosis status is presented in Table 2, while the use of different conservative treatments is presented in Supplementary Figure 1.

**Table 2:** Lipedema related variables from self-report (*n* = 432).

Lipedema diagnosis	
Yes, by health care professional	398 (92%)
Suspicion from health care professional, not confirmed diagnosis	28 (6%)
No, but have similar symptoms	5 (1%)
Missing	1 (0%)
Concomitant lymphedema	110 (25%)
Time from symptoms to diagnosis	
< 2 years	27 (6%)
2–5 years	46 (11%)
6–10 years	50 (12%)
> 10 years	306 (71%)
Missing	3 (1%)

Data presented as *n* (%).

The floor and ceiling effect, mean and standard deviations (SD) of the subscales and items, including the amount of missing data are presented in Supplementary Table 2. Ceiling effect was observed for some individual items; item 11 (44.7%), item 7 (43.6%), and item 9 (43.3%), whereas floor effects were observed for item 3b (26.5%) and item 12 (25.6%). However, none of the subscales were affected by floor or ceiling effect.

The CFA without modifications is presented in Table 3 and Supplementary Table 3, and shows CFI = 0.929, TLI = 0.922, SRMR = 0.073, and RMSEA = 0.079 (90% CI: 0.073 to 0.084). All composite reliability were considered reasonable (ranged from 0.890 to 0.916). The model yielded a chi-square statistic of *χ*^2^(344) = 1091.4. Covariance between subscales were as expected, where the correlations ranged from 0.467 to 0.600, but a larger correlation (0.819) was found between the subscales Function and Symptoms. Modification indices were evaluated, and two modifications were performed between items 5 and 8, and between items 17 and 18. The CFA with modification indices is presented in Supplementary Tables 4, 5. Model fit statistics for the LIPQOL structure validity showed CFI = 0.944, TLI = 0.938, SRMR = 0.068, and RMSEA = 0.070 (90% CI: 0.064 to 0.075). The modified model yielded a chi-square statistic of *χ*^2^(342) = 929.1

**Table 3:** Confirmatory factor analysis and composite reliability values of the LIPQOL questionnaire.

Factors/Items	Std. coeff.
Latent factor correlation (Function, Body image)	0.600
Latent factor correlation (Function, Symptoms)	0.819
Latent factor correlation (Function, Feelings)	0.554
Latent factor correlation (Body image, Symptoms)	0.573
Latent factor correlation (Body image, Feelings)	0.546
Latent factor correlation (Symptoms, Feelings)	0.467
Function (composite reliability =0.905)	
To what extent do swollen and painful legs and their treatment limit you in the following daily activities?	
Item 3a: Walking.	0.836
Item 3b: Bending forwards, e.g., when putting on shoes or performing foot care	0.653
Item 3c: Standing	0.772
Item 3d: Sitting	0.704
Item 3e: At work	0.817
Item 3f: Household chores	0.849
Item 4: How much are your leisure activities/social life affected?	0.856
Item 5: How much does wearing compression garments bother you?	0.325
Body image (composite reliability =0.916)	
Item 6: To what extent do you have difficulty finding clothes that you like to wear?	0.613
Item 7: To what extent do you believe lipedema affects your appearance?	0.790
Item 8: To what extent do you believe that wearing compression garments negatively affects your self-confidence?	0.536
Item 9: To what extent do swollen legs affect your self-image?	0.826
Item 10: To what extent does lipedema affect your self-confidence?	0.882
Item 11: To what extent do you believe lipedema affects your willingness to appear in public settings (e.g., swimming pools, dancing, etc.)?	0.734
Item 12: Does lipedema affect your romantic relationship?	0.777
Item 13: Does lipedema affect your relationships with other people?	0.876
Symptoms (composite reliability = 0.890)	
Item 14: To what extent do you experience pain due to lipedema?	0.853
Item 15: To what extent do you experience pain when pressure is applied or when touched?	0.623
Item 16: Do your legs swell during the course of the day?	0.687
Item 17: Do you experience a feeling of pressure in your legs?	0.819
Item 18: Do your legs feel heavier as the day progresses?	0.836
Item 19: Does lipedema reduce your physical performance capacity?	0.832
Feelings (composite reliability = 0.901)	
During the past week, have you experienced…	
Item 20: Sleep problems?	0.606
Item 21: Difficulties concentrating, e.g., when reading?	0.738
Item 22: Feelings of tension?	0.837
Item 23: Worries?	0.879
Item 24: Increased irritability?	0.805
Item 25: Low mood?	0.772

*N* = 351 (81% of total sample).

The sensitivity analysis carried out using FIML yielded results very much like those obtained from the complete-case ML analysis. The overall model fit remained essentially the same. In both cases the CFI was 0.873, the TLI was 0.860 with FIML as against 0.861 without FIML, and the RMSEA was 0.072 (90% CI 0.067–0.077) as compared with 0.073 (90% CI 0.067–0.078). The SRMR was 0.068 in the FIML analysis and 0.074 in the complete-case analysis.

The factor-loading estimates were generally stable as well. In the FIML analysis, the unstandardized loading for item 5 was 0.281 (95% CI 0.157–0.405, *p* < 0.001), compared with 0.225 (95% CI 0.093–0.357, *p* < 0.001) in the complete-case analysis. For the other items which had relatively high levels of missing data, the loading estimates were also similar across the different analyses. Therefore, although ML-FIML was not the estimator of choice for the ordinal indicators, the sensitivity analysis showed that excluding incomplete cases did not materially affect the CFA results. Correlation between the LIPQOL and RAND-12 is shown in Table 4. All LIPQOL variables were significantly associated with both PCS and MCS (*P* < 0.001). Large correlation was observed between PCS and Function (Spearman's rho = −0.682), Symptoms (Spearman's rho = −0.550) and overall quality of life (Spearman's rho = 0.554). Large correlation was seen between MCS and Feelings (Spearman's rho = −0.685) and overall quality of life (Spearman's rho = 0.608).

**Table 4:** Correlation between rAND-12 and LIPQOL.

	PCS		MCS	
	Spearman rho	95% CI	Spearman rho	95% CI
Item 1: To what extent are you affected by the increase in leg circumference?	−0.368	−0.447 to −0.283	−0.259	−0.345 to −0.169
Item 2: To what extent are you affected by pain in your legs?	−0.512	−0.576 to −0.435	−0.308	−0.394 to −0.222
Function	−0.682	−0.724 to −0.621	−0.471	−0.536 to −0.387
Body image	−0.348	−0.425 to −0.259	−0.422	−0.489 to −0.333
Symptoms	−0.550	−0.613 to −0.481	−0.357	−0.434 to −0.269
Feelings	−0.466	−0.530 to −0.381	−0.685	−0.731 to −0.630
Quality of life	0.554	0.478 to 0.611	0.608	0.540 to 0.661

Correlations were calculated using complete-case analysis, listwise deletion; *N* = 428 (99% of total sample). PCS: Physical component score. MCS: Mental component score. CI: Confidence interval. All *p* values were <0.001.

## 4 Discussion

The aim of this study was to validate a disease-specific questionnaire (LIPQOL) to assess QoL in females with lipedema in a Norwegian population. In line with the hypothesis, the Norwegian version of the LIPQOL questionnaire showed reasonable but not unequivocal support for the proposed four-factor structure model fit. The reliability was reasonable, and large correlation was observed between the subscales and PCS and MCS from RAND-12. No floor or ceiling effects were observed in any of the subscales.

The investigated tests of reliability of the LIPQOL was reasonable with composite reliability for all subscales ranged from 0.890 to 0.916. However, we did not measure test-retest reliability, which should be performed in future studies. The LIPQOL questionnaire showed reasonable but not unequivocal support for the proposed four-factor structure construct validity with model fit, with RMSEA of 0.079 (90% CI: 0.073 to 0.084), SRMR = 0.073 CFI = 0.929 and TLI = 0.922. Although the RMSEA was slightly higher than the ideal threshold, it remained within the reasonable range. Despite the RMSEA in the upper range, the SRMR was within the desired range. This is probably because the use of RMSEA in ordinal data may overestimate misfit (17), and SRMR may be a more suitable measure for assessing model fit in this study. Besides overall high factor loadings, one item exhibited a low factor loading (Item 5, 0.325) within the Function subscale. This item assesses whether participants perceive wearing compression garments as burdensome, which is not directly related to functional ability. Consequently, a participant may demonstrate excellent functional capacity while still finding compression garments burdensome, likely accounting for the low factor loading for this item. The low loading therefore indicates that this item contributes less to the measurement of Function than the remaining items in the subscale. While the function subscale demonstrated satisfactory overall reliability, the presence of item 5 may introduce some heterogeneity into the subscale, and scores should therefore be interpreted with awareness that not all items appear to reflect physical function to the same degree.

Moreover, the RMSEA may have been influenced by cross-loadings. Modification indices indicated covariance between item 5 and item 8. Both items address the use of compression garments, but belong in different subscales, as item 5 was in the subscale function and item 8 was in the subscale Body image. Item 5 addresses how burdensome wearing compression garments is, whereas item 8 addresses how much the negative impact of wearing compression garments on their self-confidence. This explains the observed covariance between these two items, despite the items belonging to different subscales. This provides a theoretically plausible explanation for the observed covariance, However, the covariance was identified through *post hoc* inspection of modification indices and should therefore be regarded as data-driven modification rather than *a priori* model specification. Although the shared content of the items provides a plausible substantive explanation for the association, this modification should be confirmed in independent samples. Furthermore, because the overlap occurs across two different subscales, future studies may consider whether aspects of compression garment use are relevant to both the function and body image domains. We limited the model modifications to two, as our aim was to make the minimum number of theoretically justifiable changes necessary to achieve acceptable model fit while minimizing the risk of overfitting the model.

Missing data were found in a small number of conditionally applicable items and thus constituted a possible source of selection bias in the complete-case CFA-based WLSMV. Yet the sensitivity analysis, which included the partially observed cases, yielded almost exactly the same model fit and generally similar parameter estimates. This indicates that the missing data and the consequent selection of complete cases had only a minor effect on the substantive results of the CFA. However, statistical missing-data methods such as FIML cannot completely resolve the underlying issue of item non-applicability. This should therefore be considered when interpreting missing-data patterns and when comparing findings across populations or language versions.

Moreover, Item 17 and item 18 were also highly correlated, Item 17 assesses if the participants have feelings of pressure in their legs, while item 18 assesses if the legs feel heavier throughout the day. These two items assess similar symptoms, however they differ, as item 17 assesses the presence of a symptom, while item 18 assesses worsening throughout the day. Given their conceptual overlap and placement within the same subscale, a correlation between these items is theoretically plausible. The correlation was identified through *post hoc* inspection of modification indices and subsequently judged to be substantively meaningful. Nevertheless, because this modification was data driven, confirmation in independent samples is warranted. The relatively strong associations between some of the subscales, particularly Function and Symptoms, should be considered when interpreting the instrument. However, strong associations between domains do not necessarily indicate that they measure the same construct. Symptoms and functional limitations are conceptually distinct aspects of quality of life but are also likely to be closely related in females with lipedema. The subscale scores should therefore be interpreted as related rather than independent dimensions of quality of life.

The LIPQOL questionnaire demonstrated acceptable convergent validity. The associations with the RAND-12 were consistent with our expectations, with positive correlations between the subscales related to physical function and symptoms and the PCS, and between the Feelings subscale and the MCS. These findings suggest that the disease-specific impairments captured by the LIPQOL are reflected in broader aspects of generic health-related quality of life, while the measures remain sufficiently distinct to capture different aspects of quality of life.

The removal of the explicit “Not applicable” response option represents a minor modification to the response structure of the original LIPQOL. However, because non-applicable items are excluded from the calculation of domain scores in both versions, the modification concerns how non-applicability is recorded rather than how the questionnaire is scored. It may nevertheless influence how respondents approach potentially non-applicable items and should be considered when interpreting missing responses and comparing the Norwegian and German versions.

This tool may be useful in both clinical settings and in research. It is a short questionnaire with easy scoring, which may be feasible in the clinic. Moreover, it shows preliminary support for its validity and reliability and showed reasonable psychometric properties. However, this is the first validation study of the LIPQOL questionnaire, and the German version has not yet been published or validated either.

Future research should first seek to further establish the measurement properties of the LIPQOL through continued German-Norwegian collaboration and resolve the remaining methodological issues identified in the present study. This would provide a stronger foundation for subsequent cross-cultural validation and evaluation of the instrument in other languages and populations.

This questionnaire has the potential to be a standard disease-specific questionnaire for assessing QoL in females with lipedema. The strong correlation between function and symptoms suggests that symptom severity has a substantial impact on physical functioning. This highlights the importance of symptom management for maintaining functional ability, QoL, and work capacity.

25% of the participants also had concomitant lymphedema, which may affect the results. As participants were recruited through a patient organization serving patients with both lipedema and lymphedema, the prevalence of self-reported lymphedema in our sample may be higher than that of the broader lipedema population. Furthermore, the available evidence regarding the prevalence of coexisting lymphedema among females with lipedema remains limited, and robust estimates are lacking. Moreover, patients with lymphedema seems to have more functional disability and lower life satisfaction compared to females with lipedema, although they represent similar depression rates and QoL (18). This study has both strengths and limitations. Strengths include the robust overall sample size (*n* = 432). It is also the first validation study using this questionnaire. Other validated (9) and non-validated (19) lipedema-specific questionnaires assess symptom burden; however this is the only multi-domain lipedema-specific health-related QoL instrument. Moreover, most studies use the LYMQOL questionnaire, or other generic QOL PROMS not adapted to lipedema (1).

Nevertheless, several limitations should be acknowledged. The cross-sectional design precluded assessment of test-retest reliability and responsiveness. Information on previous lipedema surgery, stage, and type was not collected, limiting assessment of measurement properties across clinically relevant subgroups. As data collection was anonymous, reported diagnoses could not be independently verified. Recruitment through the patient organization may also have introduced selection bias towards more engaged patients, potentially limiting representativeness.

Several measurement properties remain to be investigated. Cross-cultural validity and formal content validity according to COSMIN recommendations were not evaluated, and formal cognitive interviewing was not undertaken. The findings should therefore be regarded as initial evidence for the measurement properties examined. A detailed comparison of the study procedures with COSMIN recommendations (20) is provided in the Supplementary Material.

Some individual items showed floor or ceiling effects, although not at the subscale level, and their implications for responsiveness remain uncertain. Some missing responses may reflect genuine item non-applicability, particularly regarding compression-garment use and relationship status. Sensitivity analyses suggested limited influence of missing-data handling, although FIML cannot address structural non-applicability (11).

Finally, the translation used a pragmatic AI-assisted approach combined with researcher, native German-speaker, and patient review rather than formal back-translation (11). Further evaluation, particularly of cross-cultural validity and longitudinal measurement properties, is warranted.

## 5 Conclusion

The findings provide encouraging, although not unequivocal, initial support for the measurement properties of the Norwegian LIPQOL for assessing quality of life among females with lipedema. Further studies are needed to provide a more comprehensive evaluation of the instrument.

## Data Availability

The raw data supporting the conclusions of this article will be made available by the authors, without undue reservation.
