# Supplementary material for: Initial psychometric evaluation of the Lipedema Quality of Life questionnaire in females with lipedema in Norway
Source: Front Glob Womens Health. 2026 Oct 9;7:1917274. doi: 10.3389/fgwh.2026.1917274 (PMC13692784; doi:10.3389/fgwh.2026.1917274)
Supplement: Supplementary file 1 [file Table1.docx]

Supplementary file: Initial psychometric evaluation of the Lipedema Quality of Life (LIPQOL) questionnaire in females with lipedema in Norway

This supplementary file summarises the reporting of the study according to the COSMIN Study Design Checklist and COSMIN Reporting Guideline for studies on measurement properties of patient-reported outcome measures. It provides an overview of the translation process and indicates which COSMIN recommendations were addressed, modified, or not assessed in the present study.

Contents

[COSMIN Study Design checklist for Patient-reported outcome measurement instruments	2](#_Toc240442523)

[COSMIN reporting guideline for studies on measurement properties of patient-reported outcome measures	4](#_Toc240442524)

# COSMIN Study Design checklist for Patient-reported outcome measurement instruments

Mokkink, Lidwine B., et al. "COSMIN Study Design checklist for Patient-reported outcome measurement instruments." *Amsterdam, The Netherlands* 2019 (2019): 1-32.

| COSMIN item | Complies with guideline |
| --- | --- |
| TR1 Languages Report the original/source language and the target language. | Yes. The Norwegian version is also provided as an supplementary file along with the manuscript, and the translation document, which includes the German version is also provided |
| TR2 Forward/back translation Report whether forward and backward translation were performed and the number of translations. | Modified: The German LIPQOL was translated into Norwegian by three Norwegian researchers, with support from AI-based translation tools. The translation was reviewed against the original German version by a native German-speaking researcher with strong Norwegian language skills. Translation issues were discussed and resolved by consensus. The Norwegian version was subsequently reviewed by two patient representatives. |
| TR3 Forward translators: language Report whether the forward translators were native speakers of the target language. | Yes. All three forward translators were native Norwegian speakers. |
| TR4 Forward translators: expertise Report the translators' relevant clinical/construct expertise or whether they were naive to the construct. | Yes. The forward translators were health researchers with relevant clinical and research expertise, including knowledge of lipedema and patient-reported outcomes. |
| TR5 Back translators: language Report whether the back translators were native speakers of the original/source language. | Modified. No formal backward translation was performed. Instead, the complete Norwegian translation was carefully reviewed against the original German version by a native German-speaking researcher fluent in Norwegian. Translation issues were discussed with the Norwegian translators and resolved by consensus. |
| TR6 Back translators: expertise Report whether the back translators were naive to the disease and construct. | Modified. No formal back-translation was performed. The Norwegian translation was reviewed against the German original by a native German-speaking researcher fluent in Norwegian. |
| TR7 Independence Report whether translations were performed independently. | Modified. The forward translation was performed collaboratively by the three Norwegian researchers, followed by review and consensus with the German-speaking researcher. |
| TR8 Reconciliation Describe how differences between translations were resolved. | Yes. Translation issues were discussed between the Norwegian translator and the German translation examiner, and consensus was achieved. |
| TR9 Review Report whether the translation was reviewed by a committee or others, including involvement of the original developers where applicable. | Yes. The total research group including a German developer and patient representatives. |
| TR10 Documentation Report whether the translation process was documented. | Yes. The versions and correspondence was recorded, and the German and Norwegian version is provided as an supplementary to the manuscript. |
| TR11 Pilot testing Report whether the translated PROM was evaluated with patients for relevance, comprehensiveness and comprehensibility. | Yes. Two patient representatives. |
| TR12 Target population Report whether pilot testing involved patients representing the target population. | Yes. Two patient representatives. |

# COSMIN reporting guideline for studies on measurement properties of patient-reported outcome measures

Gagnier, Joel J., et al. "COSMIN reporting guideline for studies on measurement properties of patient-reported outcome measures." *Quality of life research* 30.8 (2021): 2197-2218.

Part A. General reporting recommendations

Title

| **COSMIN item** | **Complies with guideline** |
| --- | --- |
| **T1**  Title | **Yes** |

Abstract

| **COSMIN item** | **Complies with guideline** |
| --- | --- |
| **A1**  Objectives | **Yes** |
| **A2**  Design | **Yes** |
| **A3**  Methods | **Yes** |
| **A4**  Results | **Yes** |
| **A5**  Discussion/Conclusions | **Yes** |

Introduction

| **COSMIN item** | **Complies with guideline** |
| --- | --- |
| **I1**  PROM | **Yes** |
| **I2**  Target population & context of use | **Yes** |
| **I3**  State of knowledge & rationale | **Yes** |
| **I4**  Objectives | **Yes** |

General Methods

| **COSMIN item** | **Complies with guideline** |
| --- | --- |
| **GM1**  Study design | **Yes** |
| **GM2**  Participants | **Yes** |
| **GM3**  PROM details | **Yes** |
| **GM4**  Additional data collection | **Yes** |
| **GM5**  Time point procedures | **Yes** |
| **GM6**  Justification for sample size | **Yes** |
| **GM7**  Statistical analyses | **Yes** |
| **GM8**  Missing data | **Yes** |
| **GM9**  Unplanned analysis | **Yes** |

General Results

| **COSMIN item** | **Complies with guideline** |
| --- | --- |
| **GR1**  Participant characteristics | **Yes** |
| **GR2**  Sample size | **Yes** |
| **GR3**  Missing data | **Yes** |
| **GR4**  Results | **Yes** |

Discussion / Conclusions

| **COSMIN item** | **Complies with guideline** |
| --- | --- |
| **DC1**  Measurement property evidence | **Yes** |
| **DC2**  Practical relevance | **Yes** |
| **DC3**  Strengths and limitations | **Yes** |
| **DC4**  Generalizability | **Yes** |
| **DC5**  Instrument changes | **Yes** |
| **DC6**  Future research | **Yes** |
| **DC7**  Conclusions | **Yes** |

Other Information

| **COSMIN item** | **Complies with guideline** |
| --- | --- |
| **O1**  Conflict of interest | **Yes** |

Part B. Measurement-property-specific reporting recommendations

Content Validity

| **COSMIN item** | **Complies with guideline** |
| --- | --- |
| **CV1**  Relevance - Methods | The instrument was originally developed in Germany. The relevance of the Norwegian translation was considered by patients and clinician-researchers. |
| **CV2**  Comprehensiveness - Methods | Based on the original German instrument; not specifically assessed in the Norwegian study. |
| **CV3**  Comprehensibility - Methods | Considered during the Norwegian translation process by patients and clinician-researchers. |
| **CV4**  Relevance - Results | Patients and clinician-researchers considered the Norwegian translation relevant. |
| **CV5**  Comprehensiveness - Results | Not specifically assessed in the Norwegian study. |
| **CV6**  Comprehensibility - Results | Patients and clinician-researchers considered the Norwegian translation understandable. |

Structural Validity

| **COSMIN item** | **Complies with guideline** |
| --- | --- |
| **SV1**  Rationale for approach | **Yes** |
| **SV2**  Statistical analyses - Methods | **Yes** |
| **SV3**  Statistical analyses - Results | **Yes** |

Internal Consistency

| **COSMIN item** | **Complies with guideline** |
| --- | --- |
| **IC1**  Statistical analyses - Methods | **Yes** |
| **IC2**  Statistical analyses - Results | **Yes** |

Cross-Cultural Validity / Measurement Invariance

| **COSMIN item** | **Complies with guideline** |
| --- | --- |
| **CCV1**  Group variable | Cross-cultural validity/measurement invariance analyses were not performed due to sample size limitations. |
| **CCV2**  Rationale for approach | Cross-cultural validity/measurement invariance analyses were not performed due to sample size limitations. |
| **CCV3**  Statistical analyses - Methods | Cross-cultural validity/measurement invariance analyses were not performed due to sample size limitations. |
| **CCV4**  Statistical analyses - Results | Cross-cultural validity/measurement invariance analyses were not performed due to sample size limitations. |

Reliability

| **COSMIN item** | **Complies with guideline** |
| --- | --- |
| **R1**  Stability of patients - Methods | The study was cross-sectional and did not include repeated measurements. |
| **R2**  Statistical analyses - Methods | The study was cross-sectional and did not include repeated measurements. |
| **R3**  Sources of variation - Methods | The study was cross-sectional and did not include repeated measurements. |
| **R4**  Time interval - Methods | The study was cross-sectional and did not include repeated measurements. |
| **R5**  Stability of patients - Results | The study was cross-sectional and did not include repeated measurements. |
| **R6**  Statistical analyses - Results | The study was cross-sectional and did not include repeated measurements. |

Measurement Error

| **COSMIN item** | **Complies with guideline** |
| --- | --- |
| **ME1**  Stability of patients - Methods | The study was cross-sectional and did not include repeated measurements. |
| **ME2**  Statistical analyses - Methods | The study was cross-sectional and did not include repeated measurements. |
| **ME3**  Sources of variation - Methods | The study was cross-sectional and did not include repeated measurements. |
| **ME4**  Time interval - Methods | The study was cross-sectional and did not include repeated measurements. |
| **ME5**  Stability of patients - Results | The study was cross-sectional and did not include repeated measurements. |
| **ME6**  Statistical analyses - Results | The study was cross-sectional and did not include repeated measurements. |

Criterion Validity

| **COSMIN item** | **Complies with guideline** |
| --- | --- |
| **CriV1**  Criterion | **Yes** |
| **CriV2**  Statistical analyses - Methods | **Yes** |
| **CriV3**  Statistical analyses - Results | **Yes** |

Hypotheses Testing for Construct Validity

| **COSMIN item** | **Complies with guideline** |
| --- | --- |
| **ConV1**  Hypotheses - Methods | **Yes** |
| **ConV2**  Statistical analyses - Methods | **Yes** |
| **ConV3**  Statistical analyses - Results | **Yes** |

Responsiveness

| **COSMIN item** | **Complies with guideline** |
| --- | --- |
| **Resp1**  Hypotheses - Methods | The study was cross-sectional and did not include repeated measurements. |
| **Resp2**  Intervention / Exposure - Methods | The study was cross-sectional and did not include repeated measurements. |
| **Resp3**  Statistical analyses - Methods | The study was cross-sectional and did not include repeated measurements. |
| **Resp4**  Statistical analyses - Results | The study was cross-sectional and did not include repeated measurements. |
