# Supplementary material for: Initial psychometric evaluation of the Lipedema Quality of Life questionnaire in females with lipedema in Norway
Source: Front Glob Womens Health. 2026 Oct 9;7:1917274. doi: 10.3389/fgwh.2026.1917274 (PMC13692784; doi:10.3389/fgwh.2026.1917274)
Supplement: Supplementary file 2 [file Table2.docx]

| Supplementary table 2. Floor and ceiling effect subscale scores, and items scores from the LIPQOL questionnaire. | | | | | | |
| --- | --- | --- | --- | --- | --- | --- |
| Scales and items | | **Floor effect, %** | **Ceiling effect, %** | **N** | **Missing, n** | **Mean (SD)** |
| Item 1: To what extent are you affected by the increase in leg circumference? | | 2.3 % | 36.2 % | 428 | 4 | 3.1 (0.8) |
| Item 2: To what extent are you affected by pain in your legs? | | 1.2 % | 33.6 % | 429 | 3 | 3.1 (0.8) |
|  |  |  |  |  |  |  |
| Function | |  |  |  |  |  |
| Scale score | | 0.5 % | 0.7 % | 431 | 1 | 2.5 (0.6) |
| To what extent do swollen and painful legs and their treatment limit you in the following daily activities? | Item 3a: Walking | 7.2 % | 17.5 % | 429 | 3 | 2.6 (0.9) |
|  | Item 3b: Bending forwards, e.g. when putting on shoes or performing foot care | 26.5 % | 7.0 % | 430 | 2 | 2.1 (0.9) |
|  | Item 3c: Standing | 4.7 % | 20.9 % | 430 | 2 | 2.7 (0.8) |
|  | Item 3d: Sitting | 19.8 % | 6.5 % | 429 | 3 | 2.2 (0.8) |
|  | Item 3e: At work | 11.4 % | 18.1% | 386 | 46 | 2.6 (0.9) |
|  | Item 3f: Household chores | 8.8 % | 12.3 % | 430 | 2 | 2.5 (0.8) |
| Item 4: To what extent are your leisure activities / social life affected by this? | | 5.8 % | 23.0 % | 430 | 2 | 2.7 (0.9) |
| Item 5: How much does wearing compression garments bother you? | | 7.5 % | 15.7 % | 415 | 17 | 2.6 (0.8) |
|  |  |  |  |  |  |  |
| Body image | |  |  |  |  |  |
| Scale score | | 0.0% | 1.6 % | 431 | 1 | 2.8 (0.6) |
| Item 6: To what extent do you have difficulty finding clothes that you like to wear? | | 6.3 % | 21.1 % | 431 | 1 | 2.8 (0.9) |
| Item 7: To what extent do you believe lipedema affects your appearance? | | 0.7 % | 43.6 % | 431 | 1 | 3.3 (0.7) |
| Item 8: To what extent do you believe that wearing compression garments negatively affects your self‑confidence? | | 21.8 % | 13.2 % | 409 | 23 | 2.3 (1.0) |
| Item 9: To what extent do swollen legs affect your self‑image? | | 3.3 % | 43.3 % | 430 | 2 | 3.3 (0.9) |
| Item 10: To what extent does lipedema affect your self‑confidence? | | 4.4 % | 37.4 % | 431 | 1 | 3.1 (0.9) |
| Item 11: To what extent do you believe lipedema affects your willingness to appear in public settings (e.g. swimming pools, dancing, etc.)? | | 6.7 % | 44.7 % | 430 | 2 | 3.1 (1.0) |
| Item 12: Does lipedema affect your romantic relationship? | | 25.6 % | 13.2 % | 418 | 14 | 2.2 (1.0) |
| Item 13: Does lipedema affect your relationships with other people? | | 20.0 % | 7.4 % | 430 | 2 | 2.2 (0.8) |
|  |  |  |  |  |  |  |
| Symptoms | |  |  |  |  |  |
| Scale score | | 0.0 % | 5.1 % | 431 | 1 | 3.0 (0.6) |
| Item 14: To what extent do you experience pain due to lipedema? | | 0.7 % | 26.5 % | 430 | 2 | 3.0 (0.8) |
| Item 15: To what extent do you experience pain when pressure is applied or when touched? | | 1.6 % | 36.9 % | 431 | 1 | 3.1 (0.8) |
| Item 16: Do your legs swell during the course of the day? | | 1.9 % | 15.1 % | 430 | 2 | 2.7 (0.8) |
| Item 17: Do you experience a feeling of pressure in your legs? | | 0.9 % | 32.2 % | 428 | 4 | 3.0 (0.8) |
| Item 18: Do your legs feel heavier as the day progresses? | | 0.7 % | 26.7 % | 431 | 1 | 3.0 (0.7) |
| Item 19: Does lipedema reduce your physical performance capacity? | | 2.1 % | 36.3 % | 430 | 2 | 3.1 (0.8) |
|  |  |  |  |  |  |  |
| Feelings | |  |  |  |  |  |
| Scale score | | 2.6 % | 1.9 % | 431 | 1 | 2.3 (0.7) |
| During the past week, have you experienced… | Item 20: Sleep problems? | 12.5 % | 15.6 % | 431 | 1 | 2.5 (0.9) |
|  | Item 21: Difficulties concentrating, e.g. when reading? | 22.2 % | 9.4 % | 428 | 4 | 2.2 (0.9) |
|  | Item 22: Feelings of tension? | 14.2 % | 15.9 % | 429 | 3 | 2.5 (0.9) |
|  | Item 23: Worries? | 14.9 % | 17.0 % | 430 | 2 | 2.4 (0.9) |
|  | Item 24: Increased irritability? | 21.2 % | 8.4 % | 430 | 2 | 2.2 (0.9) |
|  | Item 25: Low mood? | 23.3 % | 9.3 % | 430 | 2 | 2.2 (0.9) |
|  |  |  |  |  |  |  |
| Item 26: How would you rate your current overall quality of life? | | 0.5% | 1.9% | 431 | 1 | 5.5 (1.9) |
| N=432 | | | | | | |
