# Supplementary material for: Initial psychometric evaluation of the Lipedema Quality of Life questionnaire in females with lipedema in Norway
Source: Front Glob Womens Health. 2026 Oct 9;7:1917274. doi: 10.3389/fgwh.2026.1917274 (PMC13692784; doi:10.3389/fgwh.2026.1917274)
Supplement: Supplementary file 3 [file Table3.docx]

Model fit

Chi-square test

| Model | Χ² | df | p |
| --- | --- | --- | --- |
| Baseline model | 10886.187 | 378 |  |
| Factor model | 1091.429 | 344 | < .001 |

Note. The standard error method is robust.sem.

Additional fit measures

Fit indices

| Index | Value |
| --- | --- |
| Comparative Fit Index (CFI) | 0.929 |
| Tucker-Lewis Index (TLI) | 0.922 |
| BentlerBonett Nonnormed Fit Index (NNFI) | 0.922 |
| Bentler-Bonett Normed Fit Index (NFI) | 0.900 |
| Parsimony Normed Fit Index (PNFI) | 0.886 |
| Bollen's Relative Fit Index (RFI) | 0.890 |
| Bollen's Incremental Fit Index (IFI) | 0.929 |
| Relative Noncentrality Index (RNI) | 0.929 |

Note. Except for the PNFI, the fit indices are scaled because of categorical variables in the data.

Other fit measures

| Metric | Value |
| --- | --- |
| Root mean square error of approximation (RMSEA) | 0.079 |
| RMSEA 90% CI lower bound | 0.073 |
| RMSEA 90% CI upper bound | 0.084 |
| RMSEA pvalue | 1.913×10^−13^ |
| Standardized root mean square residual (SRMR) | 0.073 |
| Hoelter's critical N (α = .05) | 138.527 |
| Hoelter's critical N (α = .01) | 145.503 |
| Goodness of fit index (GFI) | 0.979 |
| McDonald fit index (MFI) | 0.397 |
| Expected cross validation index (ECVI) |  |

Note. The RMSEA results are scaled because of categorical variables in the data.

Parameter estimates

Factor loadings

|  |  |  |  |  |  | 95% Confidence Interval | |
| --- | --- | --- | --- | --- | --- | --- | --- |
| Factor | Indicator | Std. estimate | Std. Error | z-value | p | Lower | Upper |
| Funksjon | LIPQOL_3a | 0.836 | 0.021 | 40.225 | < .001 | 0.795 | 0.877 |
|  | LIPQOL_3b | 0.653 | 0.036 | 18.162 | < .001 | 0.582 | 0.723 |
|  | LIPQOL_5 | 0.325 | 0.055 | 5.909 | < .001 | 0.217 | 0.433 |
|  | LIPQOL_3c | 0.772 | 0.028 | 27.649 | < .001 | 0.718 | 0.827 |
|  | LIPQOL_3d | 0.704 | 0.033 | 21.087 | < .001 | 0.639 | 0.770 |
|  | LIPQOL_3e | 0.817 | 0.023 | 35.571 | < .001 | 0.772 | 0.862 |
|  | LIPQOL_3f | 0.849 | 0.019 | 45.687 | < .001 | 0.813 | 0.886 |
|  | LIPQOL_4 | 0.856 | 0.024 | 35.873 | < .001 | 0.809 | 0.903 |
| Kroppsbilde | LIPQOL_6 | 0.613 | 0.043 | 14.379 | < .001 | 0.529 | 0.696 |
|  | LIPQOL_7 | 0.790 | 0.031 | 25.178 | < .001 | 0.729 | 0.852 |
|  | LIPQOL_8 | 0.536 | 0.047 | 11.481 | < .001 | 0.444 | 0.627 |
|  | LIPQOL_9 | 0.826 | 0.023 | 36.266 | < .001 | 0.782 | 0.871 |
|  | LIPQOL_10 | 0.882 | 0.021 | 41.922 | < .001 | 0.841 | 0.923 |
|  | LIPQOL_11 | 0.734 | 0.034 | 21.829 | < .001 | 0.669 | 0.800 |
|  | LIPQOL_12 | 0.777 | 0.029 | 26.423 | < .001 | 0.719 | 0.834 |
|  | LIPQOL_13 | 0.876 | 0.022 | 39.129 | < .001 | 0.832 | 0.920 |
| Symptomer | LIPQOL_14 | 0.853 | 0.021 | 40.153 | < .001 | 0.812 | 0.895 |
|  | LIPQOL_15 | 0.623 | 0.041 | 15.200 | < .001 | 0.543 | 0.704 |
|  | LIPQOL_16 | 0.687 | 0.033 | 20.849 | < .001 | 0.623 | 0.752 |
|  | LIPQOL_17 | 0.819 | 0.023 | 34.949 | < .001 | 0.773 | 0.865 |
|  | LIPQOL_18 | 0.836 | 0.023 | 36.394 | < .001 | 0.791 | 0.881 |
|  | LIPQOL_19 | 0.832 | 0.031 | 26.695 | < .001 | 0.771 | 0.893 |
| Følelser | LIPQOL_20 | 0.606 | 0.045 | 13.355 | < .001 | 0.517 | 0.695 |
|  | LIPQOL_21 | 0.738 | 0.032 | 22.950 | < .001 | 0.675 | 0.801 |
|  | LIPQOL_22 | 0.837 | 0.028 | 29.874 | < .001 | 0.782 | 0.892 |
|  | LIPQOL_23 | 0.879 | 0.021 | 42.790 | < .001 | 0.838 | 0.919 |
|  | LIPQOL_24 | 0.805 | 0.028 | 28.946 | < .001 | 0.751 | 0.860 |
|  | LIPQOL_25 | 0.772 | 0.029 | 26.946 | < .001 | 0.716 | 0.828 |

Factor variances

|  |  |  |  | 95% Confidence Interval | |
| --- | --- | --- | --- | --- | --- |
| Factor | Std. estimate | Std. Error | z-value p | Lower | Upper |
| Funksjon | 1.000 | 0.000 |  | 1.000 | 1.000 |
| Kroppsbilde | 1.000 | 0.000 |  | 1.000 | 1.000 |
| Symptomer | 1.000 | 0.000 |  | 1.000 | 1.000 |
| Følelser | 1.000 | 0.000 |  | 1.000 | 1.000 |

Factor Covariances

|  |  |  |  |  |  |  | 95% Confidence Interval | |
| --- | --- | --- | --- | --- | --- | --- | --- | --- |
|  |  |  | Std. estimate | Std. Error | z-value | p | Lower | Upper |
| Funksjon | ↔ | Kroppsbilde | 0.600 | 0.039 | 15.26 | < .001 | 0.523 | 0.677 |
| Funksjon | ↔ | Symptomer | 0.819 | 0.023 | 35.23 | < .001 | 0.773 | 0.865 |
| Funksjon | ↔ | Følelser | 0.554 | 0.043 | 12.99 | < .001 | 0.470 | 0.637 |
| Kroppsbilde | ↔ | Symptomer | 0.573 | 0.043 | 13.31 | < .001 | 0.489 | 0.657 |
| Kroppsbilde | ↔ | Følelser | 0.546 | 0.044 | 12.30 | < .001 | 0.459 | 0.633 |
| Symptomer | ↔ | Følelser | 0.467 | 0.045 | 10.33 | < .001 | 0.378 | 0.555 |

Residual variances

|  |  |  |  |  | 95% Confidence Interval | |
| --- | --- | --- | --- | --- | --- | --- |
| Indicator | Std. estimate | Std. Error | z-value | p | Lower | Upper |
| LIPQOL_3a | 0.301 | 0.035 | 8.651 | < .001 | 0.233 | 0.369 |
| LIPQOL_3b | 0.574 | 0.047 | 12.243 | < .001 | 0.482 | 0.666 |
| LIPQOL_5 | 0.894 | 0.036 | 24.958 | < .001 | 0.824 | 0.964 |
| LIPQOL_3c | 0.404 | 0.043 | 9.352 | < .001 | 0.319 | 0.488 |
| LIPQOL_3d | 0.504 | 0.047 | 10.721 | < .001 | 0.412 | 0.596 |
| LIPQOL_3e | 0.333 | 0.038 | 8.871 | < .001 | 0.259 | 0.406 |
| LIPQOL_3f | 0.279 | 0.032 | 8.820 | < .001 | 0.217 | 0.340 |
| LIPQOL_4 | 0.267 | 0.041 | 6.545 | < .001 | 0.187 | 0.347 |
| LIPQOL_6 | 0.624 | 0.052 | 11.951 | < .001 | 0.522 | 0.727 |
| LIPQOL_7 | 0.376 | 0.050 | 7.571 | < .001 | 0.278 | 0.473 |
| LIPQOL_8 | 0.713 | 0.050 | 14.256 | < .001 | 0.615 | 0.811 |
| LIPQOL_9 | 0.317 | 0.038 | 8.422 | < .001 | 0.243 | 0.391 |
| LIPQOL_10 | 0.222 | 0.037 | 5.975 | < .001 | 0.149 | 0.295 |
| LIPQOL_11 | 0.461 | 0.049 | 9.318 | < .001 | 0.364 | 0.557 |
| LIPQOL_12 | 0.397 | 0.046 | 8.691 | < .001 | 0.307 | 0.486 |
| LIPQOL_13 | 0.233 | 0.039 | 5.943 | < .001 | 0.156 | 0.310 |
| LIPQOL_14 | 0.272 | 0.036 | 7.507 | < .001 | 0.201 | 0.343 |
| LIPQOL_15 | 0.612 | 0.051 | 11.969 | < .001 | 0.511 | 0.712 |
| LIPQOL_16 | 0.528 | 0.045 | 11.645 | < .001 | 0.439 | 0.616 |
| LIPQOL_17 | 0.330 | 0.038 | 8.590 | < .001 | 0.254 | 0.405 |
| LIPQOL_18 | 0.301 | 0.038 | 7.842 | < .001 | 0.226 | 0.376 |
| LIPQOL_19 | 0.308 | 0.052 | 5.954 | < .001 | 0.207 | 0.410 |
| LIPQOL_20 | 0.633 | 0.055 | 11.514 | < .001 | 0.525 | 0.741 |
| LIPQOL_21 | 0.455 | 0.047 | 9.596 | < .001 | 0.362 | 0.548 |
| LIPQOL_22 | 0.299 | 0.047 | 6.368 | < .001 | 0.207 | 0.391 |
| LIPQOL_23 | 0.228 | 0.036 | 6.323 | < .001 | 0.157 | 0.299 |
| LIPQOL_24 | 0.351 | 0.045 | 7.843 | < .001 | 0.264 | 0.439 |
| LIPQOL_25 | 0.404 | 0.044 | 9.133 | < .001 | 0.317 | 0.491 |

Thresholds

|  |  |  |  |  |  | 95% Confidence Interval | |
| --- | --- | --- | --- | --- | --- | --- | --- |
| Indicator | Threshold | Estimate | Std. Error | z-value | p | Lower | Upper |
| LIPQOL_3a | t1 | −1.429 | 0.099 | −14.496 | 0.000 | −1.622 | −1.236 |
|  | t2 | −0.018 | 0.067 | −0.266 | 0.790 | −0.149 | 0.113 |
|  | t3 | 0.954 | 0.079 | 12.061 | 0.000 | 0.799 | 1.109 |
| LIPQOL_3b | t1 | −0.556 | 0.071 | −7.871 | 3.553×10^−15^ | −0.695 | −0.418 |
|  | t2 | 0.668 | 0.073 | 9.210 | 0.000 | 0.526 | 0.810 |
|  | t3 | 1.491 | 0.102 | 14.589 | 0.000 | 1.291 | 1.691 |
| LIPQOL_5 | t1 | −1.470 | 0.101 | −14.563 | 0.000 | −1.668 | −1.272 |
|  | t2 | −0.046 | 0.067 | −0.691 | 0.490 | −0.177 | 0.085 |
|  | t3 | 1.048 | 0.082 | 12.780 | 0.000 | 0.887 | 1.209 |
| LIPQOL_3c | t1 | −1.635 | 0.112 | −14.611 | 0.000 | −1.855 | −1.416 |
|  | t2 | −0.168 | 0.067 | −2.497 | 0.013 | −0.299 | −0.036 |
|  | t3 | 0.838 | 0.076 | 11.013 | 0.000 | 0.689 | 0.987 |
| LIPQOL_3d | t1 | −0.779 | 0.075 | −10.421 | 0.000 | −0.925 | −0.632 |
|  | t2 | 0.507 | 0.070 | 7.246 | 4.301×10^−13^ | 0.370 | 0.645 |
|  | t3 | 1.449 | 0.100 | 14.531 | 0.000 | 1.254 | 1.645 |
| LIPQOL_3e | t1 | −1.180 | 0.087 | −13.598 | 0.000 | −1.350 | −1.010 |
|  | t2 | 0.039 | 0.067 | 0.585 | 0.559 | −0.092 | 0.170 |
|  | t3 | 0.900 | 0.078 | 11.591 | 0.000 | 0.748 | 1.052 |
| LIPQOL_3f | t1 | −1.303 | 0.092 | −14.146 | 0.000 | −1.483 | −1.122 |
|  | t2 | 0.197 | 0.067 | 2.921 | 0.003 | 0.065 | 0.328 |
|  | t3 | 1.166 | 0.086 | 13.521 | 0.000 | 0.997 | 1.335 |
| LIPQOL_4 | t1 | −1.559 | 0.107 | −14.633 | 0.000 | −1.768 | −1.350 |
|  | t2 | −0.211 | 0.067 | −3.134 | 0.002 | −0.343 | −0.079 |
|  | t3 | 0.779 | 0.075 | 10.421 | 0.000 | 0.632 | 0.925 |
| LIPQOL_6 | t1 | −1.470 | 0.101 | −14.563 | 0.000 | −1.668 | −1.272 |
|  | t2 | −0.321 | 0.068 | −4.722 | 2.334×10^−6^ | −0.455 | −0.188 |
|  | t3 | 0.808 | 0.075 | 10.718 | 0.000 | 0.660 | 0.955 |
| LIPQOL_7 | t1 | −2.532 | 0.248 | −10.229 | 0.000 | −3.018 | −2.047 |
|  | t2 | −1.086 | 0.083 | −13.037 | 0.000 | −1.249 | −0.922 |
|  | t3 | 0.146 | 0.067 | 2.178 | 0.029 | 0.015 | 0.278 |
| LIPQOL_8 | t1 | −0.828 | 0.076 | −10.915 | 0.000 | −0.976 | −0.679 |
|  | t2 | 0.314 | 0.068 | 4.616 | 3.904×10^−6^ | 0.181 | 0.447 |
|  | t3 | 1.125 | 0.085 | 13.284 | 0.000 | 0.959 | 1.291 |
| LIPQOL_9 | t1 | −1.864 | 0.132 | −14.133 | 0.000 | −2.122 | −1.605 |
|  | t2 | −0.759 | 0.074 | −10.222 | 0.000 | −0.905 | −0.614 |
|  | t3 | 0.146 | 0.067 | 2.178 | 0.029 | 0.015 | 0.278 |
| LIPQOL_10 | t1 | −1.789 | 0.125 | −14.355 | 0.000 | −2.033 | −1.545 |
|  | t2 | −0.607 | 0.071 | −8.492 | 0.000 | −0.747 | −0.467 |
|  | t3 | 0.307 | 0.068 | 4.511 | 6.462×10^−6^ | 0.173 | 0.440 |
| LIPQOL_11 | t1 | −1.559 | 0.107 | −14.633 | 0.000 | −1.768 | −1.350 |
|  | t2 | −0.641 | 0.072 | −8.903 | 0.000 | −0.783 | −0.500 |
|  | t3 | 0.153 | 0.067 | 2.285 | 0.022 | 0.022 | 0.285 |
| LIPQOL_12 | t1 | −0.668 | 0.073 | −9.210 | 0.000 | −0.810 | −0.526 |
|  | t2 | 0.467 | 0.070 | 6.723 | 1.783×10^−11^ | 0.331 | 0.604 |
|  | t3 | 1.125 | 0.085 | 13.284 | 0.000 | 0.959 | 1.291 |
| LIPQOL_13 | t1 | −0.879 | 0.077 | −11.400 | 0.000 | −1.030 | −0.728 |
|  | t2 | 0.507 | 0.070 | 7.246 | 4.301×10^−13^ | 0.370 | 0.645 |
|  | t3 | 1.449 | 0.100 | 14.531 | 0.000 | 1.254 | 1.645 |
| LIPQOL_14 | t1 | −2.387 | 0.212 | −11.276 | 0.000 | −2.802 | −1.972 |
|  | t2 | −0.524 | 0.070 | −7.454 | 9.037×10^−14^ | −0.661 | −0.386 |
|  | t3 | 0.650 | 0.072 | 9.006 | 0.000 | 0.509 | 0.792 |
| LIPQOL_15 | t1 | −2.057 | 0.155 | −13.308 | 0.000 | −2.360 | −1.754 |
|  | t2 | −0.668 | 0.073 | −9.210 | 0.000 | −0.810 | −0.526 |
|  | t3 | 0.344 | 0.068 | 5.039 | 4.678×10^−7^ | 0.210 | 0.478 |
| LIPQOL_16 | t1 | −2.120 | 0.163 | −12.972 | 0.000 | −2.440 | −1.800 |
|  | t2 | −0.075 | 0.067 | −1.116 | 0.264 | −0.206 | 0.056 |
|  | t3 | 1.048 | 0.082 | 12.780 | 0.000 | 0.887 | 1.209 |
| LIPQOL_17 | t1 | −2.279 | 0.190 | −12.005 | 0.000 | −2.651 | −1.907 |
|  | t2 | −0.598 | 0.071 | −8.388 | 0.000 | −0.738 | −0.459 |
|  | t3 | 0.444 | 0.069 | 6.408 | 1.473×10^−10^ | 0.308 | 0.579 |
| LIPQOL_18 | t1 | −2.387 | 0.212 | −11.276 | 0.000 | −2.802 | −1.972 |
|  | t2 | −0.633 | 0.072 | −8.801 | 0.000 | −0.774 | −0.492 |
|  | t3 | 0.590 | 0.071 | 8.285 | 2.220×10^−16^ | 0.450 | 0.729 |
| LIPQOL_19 | t1 | −2.057 | 0.155 | −13.308 | 0.000 | −2.360 | −1.754 |
|  | t2 | −0.650 | 0.072 | −9.006 | 0.000 | −0.792 | −0.509 |
|  | t3 | 0.336 | 0.068 | 4.933 | 8.078×10^−7^ | 0.203 | 0.470 |
| LIPQOL_20 | t1 | −1.060 | 0.082 | −12.867 | 0.000 | −1.222 | −0.899 |
|  | t2 | 0.168 | 0.067 | 2.497 | 0.013 | 0.036 | 0.299 |
|  | t3 | 1.012 | 0.081 | 12.516 | 0.000 | 0.853 | 1.170 |
| LIPQOL_21 | t1 | −0.818 | 0.076 | −10.817 | 0.000 | −0.966 | −0.669 |
|  | t2 | 0.299 | 0.068 | 4.405 | 1.058×10^−5^ | 0.166 | 0.432 |
|  | t3 | 1.303 | 0.092 | 14.146 | 0.000 | 1.122 | 1.483 |
| LIPQOL_22 | t1 | −1.036 | 0.082 | −12.693 | 0.000 | −1.196 | −0.876 |
|  | t2 | 0.132 | 0.067 | 1.966 | 0.049 | 4.043×10^−4^ | 0.263 |
|  | t3 | 0.977 | 0.080 | 12.245 | 0.000 | 0.821 | 1.133 |
| LIPQOL_23 | t1 | −1.036 | 0.082 | −12.693 | 0.000 | −1.196 | −0.876 |
|  | t2 | 0.204 | 0.067 | 3.028 | 0.002 | 0.072 | 0.336 |
|  | t3 | 0.900 | 0.078 | 11.591 | 0.000 | 0.748 | 1.052 |
| LIPQOL_24 | t1 | −0.808 | 0.075 | −10.718 | 0.000 | −0.955 | −0.660 |
|  | t2 | 0.548 | 0.071 | 7.767 | 7.994×10^−15^ | 0.410 | 0.687 |
|  | t3 | 1.354 | 0.095 | 14.315 | 0.000 | 1.169 | 1.540 |
| LIPQOL_25 | t1 | −0.722 | 0.074 | −9.820 | 0.000 | −0.866 | −0.578 |
|  | t2 | 0.499 | 0.070 | 7.141 | 9.244×10^−13^ | 0.362 | 0.636 |
|  | t3 | 1.337 | 0.094 | 14.261 | 0.000 | 1.153 | 1.520 |
|  |  |  |  |  |  |  |  |

Reliability

|  | Coefficient ω | Coefficient α |
| --- | --- | --- |
| Funksjon | 0.910 | 0.861 |
| Kroppsbilde | 0.905 | 0.869 |
| Symptomer | 0.899 | 0.843 |
| Følelser | 0.906 | 0.859 |
| total | 0.971 | 0.930 |

Modification Indices

Cross-loadings

|  |  |  | Mod. Ind. | EPC |
| --- | --- | --- | --- | --- |
| Kroppsbilde | → | LIPQOL_5 | 103.355 | −0.767 |
| Funksjon | → | LIPQOL_19 | 87.916 | −1.119 |
| Kroppsbilde | → | LIPQOL_19 | 54.255 | −0.540 |
| Kroppsbilde | → | LIPQOL_4 | 43.818 | −0.447 |
| Følelser | → | LIPQOL_19 | 39.842 | −0.453 |
| Symptomer | → | LIPQOL_20 | 35.809 | −0.254 |
| Funksjon | → | LIPQOL_14 | 34.220 | −0.647 |
| Funksjon | → | LIPQOL_9 | 31.911 | 0.291 |
| Følelser | → | LIPQOL_9 | 31.255 | 0.426 |
| Funksjon | → | LIPQOL_20 | 31.249 | −0.259 |
| Funksjon | → | LIPQOL_17 | 27.880 | 0.594 |
| Funksjon | → | LIPQOL_18 | 26.711 | 0.582 |
| Følelser | → | LIPQOL_4 | 23.935 | −0.338 |
| Symptomer | → | LIPQOL_25 | 22.886 | 0.204 |
| Funksjon | → | LIPQOL_16 | 20.356 | 0.522 |
| Funksjon | → | LIPQOL_25 | 19.711 | 0.201 |
| Symptomer | → | LIPQOL_9 | 17.983 | 0.212 |
| Følelser | → | LIPQOL_16 | 15.956 | 0.293 |
| Symptomer | → | LIPQOL_8 | 15.798 | 0.202 |
| Kroppsbilde | → | LIPQOL_3f | 15.779 | 0.287 |
| Følelser | → | LIPQOL_14 | 14.489 | −0.268 |
| Kroppsbilde | → | LIPQOL_18 | 14.199 | 0.282 |
| Funksjon | → | LIPQOL_8 | 13.807 | 0.191 |
| Funksjon | → | LIPQOL_13 | 11.394 | −0.166 |
| Følelser | → | LIPQOL_3a | 11.188 | 0.247 |
| Følelser | → | LIPQOL_12 | 11.144 | −0.240 |
| Funksjon | → | LIPQOL_12 | 10.753 | −0.161 |
| Følelser | → | LIPQOL_17 | 10.613 | 0.238 |
| Symptomer | → | LIPQOL_13 | 9.864 | −0.154 |
| Funksjon | → | LIPQOL_10 | 9.106 | 0.153 |
| Symptomer | → | LIPQOL_10 | 8.392 | 0.145 |
| Symptomer | → | LIPQOL_22 | 7.989 | −0.117 |
| Symptomer | → | LIPQOL_24 | 7.919 | 0.119 |
| Følelser | → | LIPQOL_5 | 7.883 | −0.212 |
| Kroppsbilde | → | LIPQOL_17 | 7.836 | 0.210 |
| Følelser | → | LIPQOL_13 | 7.251 | −0.196 |
| Kroppsbilde | → | LIPQOL_20 | 7.229 | −0.184 |
| Funksjon | → | LIPQOL_24 | 7.095 | 0.120 |
| Funksjon | → | LIPQOL_6 | 7.004 | −0.133 |
| Kroppsbilde | → | LIPQOL_24 | 6.742 | 0.174 |
| Symptomer | → | LIPQOL_12 | 6.544 | −0.126 |
| Symptomer | → | LIPQOL_3a | 6.361 | −0.255 |
| Kroppsbilde | → | LIPQOL_25 | 5.972 | 0.159 |
| Følelser | → | LIPQOL_18 | 5.922 | 0.170 |
| Kroppsbilde | → | LIPQOL_3e | 5.786 | 0.174 |
| Funksjon | → | LIPQOL_22 | 5.490 | −0.104 |
| Kroppsbilde | → | LIPQOL_16 | 4.930 | 0.169 |
| Følelser | → | LIPQOL_8 | 4.822 | 0.164 |
| Symptomer | → | LIPQOL_7 | 4.784 | −0.109 |
| Symptomer | → | LIPQOL_3f | 4.373 | 0.219 |
| Kroppsbilde | → | LIPQOL_3a | 4.051 | 0.148 |

| Residual covariances | | | | |
| --- | --- | --- | --- | --- |
|  |  |  | Mod. Ind. | EPC |
| LIPQOL_5 | ↔ | LIPQOL_8 | 128.839 | −0.466 |
| LIPQOL_17 | ↔ | LIPQOL_18 | 51.266 | −0.230 |
| LIPQOL_9 | ↔ | LIPQOL_10 | 34.792 | −0.183 |
| LIPQOL_24 | ↔ | LIPQOL_25 | 31.998 | −0.197 |
| LIPQOL_16 | ↔ | LIPQOL_18 | 28.796 | −0.202 |
| LIPQOL_18 | ↔ | LIPQOL_19 | 26.383 | 0.271 |
| LIPQOL_17 | ↔ | LIPQOL_19 | 18.699 | 0.214 |
| LIPQOL_14 | ↔ | LIPQOL_19 | 18.179 | 0.201 |
| LIPQOL_4 | ↔ | LIPQOL_19 | 17.785 | −0.145 |
| LIPQOL_12 | ↔ | LIPQOL_13 | 15.603 | −0.141 |
| LIPQOL_5 | ↔ | LIPQOL_7 | 13.859 | −0.211 |
| LIPQOL_7 | ↔ | LIPQOL_13 | 13.136 | 0.187 |
| LIPQOL_14 | ↔ | LIPQOL_20 | 12.989 | −0.189 |
| LIPQOL_7 | ↔ | LIPQOL_19 | 12.263 | −0.168 |
| LIPQOL_3a | ↔ | LIPQOL_19 | 12.061 | −0.134 |
| LIPQOL_5 | ↔ | LIPQOL_3f | 11.469 | 0.199 |
| LIPQOL_20 | ↔ | LIPQOL_23 | 10.927 | 0.175 |
| LIPQOL_6 | ↔ | LIPQOL_7 | 10.926 | −0.148 |
| LIPQOL_16 | ↔ | LIPQOL_17 | 10.508 | −0.131 |
| LIPQOL_23 | ↔ | LIPQOL_25 | 10.470 | −0.116 |
| LIPQOL_7 | ↔ | LIPQOL_12 | 9.991 | 0.173 |
| LIPQOL_3f | ↔ | LIPQOL_9 | 9.861 | 0.188 |
| LIPQOL_4 | ↔ | LIPQOL_6 | 9.629 | −0.144 |
| LIPQOL_5 | ↔ | LIPQOL_6 | 9.606 | −0.175 |
| LIPQOL_16 | ↔ | LIPQOL_19 | 9.161 | 0.190 |
| LIPQOL_8 | ↔ | LIPQOL_9 | 8.778 | −0.144 |
| LIPQOL_9 | ↔ | LIPQOL_12 | 8.747 | 0.149 |
| LIPQOL_9 | ↔ | LIPQOL_13 | 8.382 | 0.140 |
| LIPQOL_4 | ↔ | LIPQOL_7 | 8.349 | −0.128 |
| LIPQOL_20 | ↔ | LIPQOL_24 | 8.271 | 0.154 |
| LIPQOL_21 | ↔ | LIPQOL_25 | 8.062 | 0.139 |
| LIPQOL_4 | ↔ | LIPQOL_13 | 8.009 | −0.120 |
| LIPQOL_3b | ↔ | LIPQOL_6 | 7.736 | −0.145 |
| LIPQOL_10 | ↔ | LIPQOL_13 | 7.713 | 0.117 |
| LIPQOL_4 | ↔ | LIPQOL_12 | 7.601 | −0.126 |
| LIPQOL_6 | ↔ | LIPQOL_12 | 7.239 | 0.150 |
| LIPQOL_22 | ↔ | LIPQOL_25 | 7.225 | 0.123 |
| LIPQOL_3e | ↔ | LIPQOL_3f | 6.944 | −0.081 |
| LIPQOL_3c | ↔ | LIPQOL_4 | 6.806 | 0.111 |
| LIPQOL_10 | ↔ | LIPQOL_12 | 6.683 | 0.119 |
| LIPQOL_3f | ↔ | LIPQOL_17 | 6.658 | 0.120 |
| LIPQOL_17 | ↔ | LIPQOL_25 | 6.554 | 0.159 |
| LIPQOL_3f | ↔ | LIPQOL_8 | 6.453 | 0.146 |
| LIPQOL_8 | ↔ | LIPQOL_18 | 6.418 | 0.155 |
| LIPQOL_3f | ↔ | LIPQOL_10 | 6.410 | 0.146 |
| LIPQOL_3e | ↔ | LIPQOL_9 | 6.395 | 0.152 |
| LIPQOL_3d | ↔ | LIPQOL_9 | 6.394 | 0.150 |
| LIPQOL_3d | ↔ | LIPQOL_3e | 6.236 | −0.090 |
| LIPQOL_6 | ↔ | LIPQOL_19 | 6.205 | −0.125 |
| LIPQOL_3a | ↔ | LIPQOL_14 | 6.034 | −0.092 |
| LIPQOL_3f | ↔ | LIPQOL_18 | 5.976 | 0.117 |
| LIPQOL_3e | ↔ | LIPQOL_4 | 5.919 | 0.095 |
| LIPQOL_14 | ↔ | LIPQOL_22 | 5.848 | −0.122 |
| LIPQOL_13 | ↔ | LIPQOL_19 | 5.771 | −0.118 |
| LIPQOL_5 | ↔ | LIPQOL_9 | 5.711 | −0.139 |
| LIPQOL_21 | ↔ | LIPQOL_23 | 5.652 | 0.110 |
| LIPQOL_19 | ↔ | LIPQOL_22 | 5.643 | −0.120 |
| LIPQOL_3f | ↔ | LIPQOL_14 | 5.622 | −0.096 |
| LIPQOL_12 | ↔ | LIPQOL_19 | 5.383 | −0.117 |
| LIPQOL_3d | ↔ | LIPQOL_4 | 5.376 | 0.114 |
| LIPQOL_3d | ↔ | LIPQOL_8 | 5.214 | 0.126 |
| LIPQOL_15 | ↔ | LIPQOL_23 | 5.122 | 0.140 |
| LIPQOL_7 | ↔ | LIPQOL_9 | 4.850 | −0.091 |
| LIPQOL_3a | ↔ | LIPQOL_10 | 4.837 | 0.125 |
| LIPQOL_9 | ↔ | LIPQOL_22 | 4.829 | 0.124 |
| LIPQOL_5 | ↔ | LIPQOL_3e | 4.802 | 0.126 |
| LIPQOL_5 | ↔ | LIPQOL_3c | 4.677 | 0.125 |
| LIPQOL_8 | ↔ | LIPQOL_14 | 4.664 | 0.124 |
| LIPQOL_3d | ↔ | LIPQOL_19 | 4.645 | 0.117 |
| LIPQOL_19 | ↔ | LIPQOL_21 | 4.568 | −0.115 |
| LIPQOL_7 | ↔ | LIPQOL_25 | 4.454 | 0.128 |
| LIPQOL_3a | ↔ | LIPQOL_5 | 4.401 | 0.130 |
| LIPQOL_3a | ↔ | LIPQOL_3b | 4.350 | −0.089 |
| LIPQOL_21 | ↔ | LIPQOL_22 | 4.335 | −0.083 |
| LIPQOL_16 | ↔ | LIPQOL_23 | 4.312 | 0.130 |
| LIPQOL_7 | ↔ | LIPQOL_15 | 4.128 | −0.111 |
| LIPQOL_3a | ↔ | LIPQOL_8 | 4.054 | 0.119 |
| LIPQOL_17 | ↔ | LIPQOL_23 | 3.982 | 0.119 |
| LIPQOL_3e | ↔ | LIPQOL_14 | 3.959 | −0.079 |
| LIPQOL_20 | ↔ | LIPQOL_21 | 3.921 | −0.089 |
| LIPQOL_18 | ↔ | LIPQOL_25 | 3.874 | 0.116 |
|  |  |  |  |  |
