# Supplementary material for: Initial psychometric evaluation of the Lipedema Quality of Life questionnaire in females with lipedema in Norway
Source: Front Glob Womens Health. 2026 Oct 9;7:1917274. doi: 10.3389/fgwh.2026.1917274 (PMC13692784; doi:10.3389/fgwh.2026.1917274)
Supplement: Supplementary file 4 [file Table4.docx]

| Supplementary Table 4. Confirmatory factor analysis and Composite Reliability values of the LIPQOL questionnaire with modification indices. | |
| --- | --- |
|  | Std. coeff. |
| Latent factor correlation (Function, Body image) | 0.593 |
| Latent factor correlation (Function, Symptoms) | 0.841 |
| Latent factor correlation (Function, Feelings) | 0.554 |
| Latent factor correlation (Body image, Symptoms) | 0.590 |
| Latent factor correlation (Body image, Feelings) | 0.547 |
| Latent factor correlation (Symptoms, Feelings) | 0.481 |
| Function (composite reliability = 0.904) |  |
| To what extent do swollen and painful legs and their treatment limit you in the following daily activities? |  |
| Item 3a: Walking. | 0.837 |
| Item 3b: Bending forwards, e.g. when putting on shoes or performing foot care | 0.654 |
| Item 3c: Standing | 0.773 |
| Item 3d: Sitting | 0.706 |
| Item 3e: At work | 0.818 |
| Item 3f: Household chores | 0.851 |
| Item 4: How much are your leisure activities / social life affected? | 0.856 |
| Item 5: How much does wearing compression garments bother you? | 0.295 |
| Body image (composite reliability = 0.915) |  |
| Item 6: To what extent do you have difficulty finding clothes that you like to wear? | 0.614 |
| Item 7: To what extent do you believe lipedema affects your appearance? | 0.791 |
| Item 8: To what extent do you believe that wearing compression garments negatively affects your self‑confidence? | 0.519 |
| Item 9: To what extent do swollen legs affect your self‑image? | 0.827 |
| Item 10: To what extent does lipedema affect your self‑confidence? | 0.883 |
| Item 11: To what extent do you believe lipedema affects your willingness to appear in public settings (e.g. swimming pools, dancing, etc.)? | 0.735 |
| Item 12: Does lipedema affect your romantic relationship? | 0.778 |
| Item 13: Does lipedema affect your relationships with other people? | 0.877 |
| Symptoms (composite reliability = 0.890) |  |
| Item 14: To what extent do you experience pain due to lipedema? | 0.857 |
| Item 15: To what extent do you experience pain when pressure is applied or when touched? | 0.622 |
| Item 16: Do your legs swell during the course of the day? | 0.688 |
| Item 17: Do you experience a feeling of pressure in your legs? | 0.759 |
| Item 18: Do your legs feel heavier as the day progresses? | 0.774 |
| Item 19: Does lipedema reduce your physical performance capacity? | 0.828 |
| Feelings (composite reliability = 0.901) |  |
| During the past week, have you experienced… |  |
| Item 20: Sleep problems? | 0.606 |
| Item 21: Difficulties concentrating, e.g. when reading? | 0.738 |
| Item 22: Feelings of tension? | 0.837 |
| Item 23: Worries? | 0.878 |
| Item 24: Increased irritability? | 0.805 |
| Item 25: Low mood? | 0.772 |
| N=351 (81% of total sample). | |
