# Supplementary material for: Initial psychometric evaluation of the Lipedema Quality of Life questionnaire in females with lipedema in Norway
Source: Front Glob Womens Health. 2026 Oct 9;7:1917274. doi: 10.3389/fgwh.2026.1917274 (PMC13692784; doi:10.3389/fgwh.2026.1917274)
Supplement: Supplementary file 5 [file Table5.docx]

Chi-square test

| Model | Χ² | df | p |
| --- | --- | --- | --- |
| Baseline model | 10886.187 | 378 |  |
| Factor model | 929.116 | 342 | < .001 |

Note. The standard error method is robust.sem.

Fit indices

| Index | Value |
| --- | --- |
| Comparative Fit Index (CFI) | 0.944 |
| Tucker-Lewis Index (TLI) | 0.938 |
| Bentler Bonett Non-normed Fit Index (NNFI) | 0.938 |
| Bentler-Bonett Normed Fit Index (NFI) | 0.915 |
| Parsimony Normed Fit Index (PNFI) | 0.885 |
| Bollen's Relative Fit Index (RFI) | 0.906 |
| Bollen's Incremental Fit Index (IFI) | 0.944 |
| Relative Noncentrality Index (RNI) | 0.944 |

Note. Except for the PNFI, the fit indices are scaled because of categorical variables in the data.

Other fit measures

| Metric | Value |
| --- | --- |
| Root mean square error of approximation (RMSEA) | 0.070 |
| RMSEA 90% CI lower bound | 0.064 |
| RMSEA 90% CI upper bound | 0.075 |
| RMSEA p value | 1.854×10^−9^ |
| Standardized root mean square residual (SRMR) | 0.068 |
| Hoelter's critical N (α = .05) | 168.189 |
| Hoelter's critical N (α = .01) | 176.693 |
| Goodness of  fit index (GFI) | 0.983 |
| McDonald fit index (MFI) | 0.512 |
| Expected cross validation index (ECVI) |  |

Note. The RMSEA results are scaled because of categorical variables in the data.

Parameter estimates

Factor loadings

95% Confidence Interval

| Factor | Indicator | Std. estimate | Std. Error | z-value | p | Lower | Upper |
| --- | --- | --- | --- | --- | --- | --- | --- |
| Funksjon | LIPQOL_3a | 0.837 | 0.021 | 40.301 | < .001 | 0.797 | 0.878 |
|  | LIPQOL_3b | 0.654 | 0.036 | 18.191 | < .001 | 0.583 | 0.724 |
|  | LIPQOL_5 | 0.295 | 0.056 | 5.244 | < .001 | 0.185 | 0.405 |
|  | LIPQOL_3c | 0.773 | 0.028 | 27.697 | < .001 | 0.719 | 0.828 |
|  | LIPQOL_3d | 0.706 | 0.033 | 21.106 | < .001 | 0.640 | 0.771 |
|  | LIPQOL_3e | 0.818 | 0.023 | 35.587 | < .001 | 0.773 | 0.863 |
|  | LIPQOL_3f | 0.851 | 0.019 | 45.923 | < .001 | 0.815 | 0.887 |
|  | LIPQOL_4 | 0.856 | 0.024 | 35.842 | < .001 | 0.809 | 0.903 |
| Kroppsbilde | LIPQOL_6 | 0.614 | 0.043 | 14.377 | < .001 | 0.530 | 0.697 |
|  | LIPQOL_7 | 0.791 | 0.031 | 25.197 | < .001 | 0.730 | 0.853 |
|  | LIPQOL_8 | 0.519 | 0.047 | 10.977 | < .001 | 0.426 | 0.612 |
|  | LIPQOL_9 | 0.827 | 0.023 | 36.299 | < .001 | 0.782 | 0.872 |
|  | LIPQOL_10 | 0.883 | 0.021 | 41.991 | < .001 | 0.842 | 0.924 |
|  | LIPQOL_11 | 0.735 | 0.034 | 21.842 | < .001 | 0.669 | 0.801 |
|  | LIPQOL_12 | 0.778 | 0.029 | 26.451 | < .001 | 0.720 | 0.835 |
|  | LIPQOL_13 | 0.877 | 0.022 | 39.195 | < .001 | 0.833 | 0.921 |
| Symptomer | LIPQOL_14 | 0.857 | 0.021 | 39.906 | < .001 | 0.814 | 0.899 |
|  | LIPQOL_15 | 0.622 | 0.041 | 15.118 | < .001 | 0.541 | 0.702 |
|  | LIPQOL_16 | 0.688 | 0.034 | 20.469 | < .001 | 0.622 | 0.754 |
|  | LIPQOL_17 | 0.759 | 0.029 | 25.894 | < .001 | 0.701 | 0.816 |
|  | LIPQOL_18 | 0.774 | 0.030 | 25.964 | < .001 | 0.716 | 0.833 |
|  | LIPQOL_19 | 0.828 | 0.031 | 26.625 | < .001 | 0.767 | 0.889 |
| Følelser | LIPQOL_20 | 0.606 | 0.045 | 13.358 | < .001 | 0.517 | 0.695 |
|  | LIPQOL_21 | 0.738 | 0.032 | 22.960 | < .001 | 0.675 | 0.801 |
|  | LIPQOL_22 | 0.837 | 0.028 | 29.865 | < .001 | 0.782 | 0.892 |
|  | LIPQOL_23 | 0.878 | 0.021 | 42.766 | < .001 | 0.838 | 0.919 |
|  | LIPQOL_24 | 0.805 | 0.028 | 28.946 | < .001 | 0.751 | 0.860 |
|  | LIPQOL_25 | 0.772 | 0.029 | 26.968 | < .001 | 0.716 | 0.828 |

Factor variances

95% Confidence Interval

| Factor | Std. estimate | Std. Error | z-value p | Lower | Upper |
| --- | --- | --- | --- | --- | --- |
| Funksjon | 1.000 | 0.000 |  | 1.000 | 1.000 |
| Kroppsbilde | 1.000 | 0.000 |  | 1.000 | 1.000 |
| Symptomer | 1.000 | 0.000 |  | 1.000 | 1.000 |
| Følelser | 1.000 | 0.000 |  | 1.000 | 1.000 |

Factor Covariances

95% Confidence Interval

|  |  |  | Std. estimate | Std. Error | z-value | p | Lower | Upper |
| --- | --- | --- | --- | --- | --- | --- | --- | --- |
| Funksjon | ↔ | Kroppsbilde | 0.593 | 0.040 | 14.99 | < .001 | 0.516 | 0.671 |
| Funksjon | ↔ | Symptomer | 0.841 | 0.023 | 37.04 | < .001 | 0.797 | 0.886 |
| Funksjon | ↔ | Følelser | 0.554 | 0.043 | 12.98 | < .001 | 0.470 | 0.638 |
| Kroppsbilde | ↔ | Symptomer | 0.590 | 0.044 | 13.48 | < .001 | 0.504 | 0.676 |
| Kroppsbilde | ↔ | Følelser | 0.547 | 0.044 | 12.31 | < .001 | 0.460 | 0.634 |
| Symptomer | ↔ | Følelser | 0.481 | 0.046 | 10.46 | < .001 | 0.391 | 0.571 |

Residual variances

95% Confidence Interval

| Indicator | Std. estimate | Std. Error | z-value | p | Lower | Upper |
| --- | --- | --- | --- | --- | --- | --- |
| LIPQOL_3a | 0.299 | 0.035 | 8.590 | < .001 | 0.231 | 0.367 |
| LIPQOL_3b | 0.572 | 0.047 | 12.178 | < .001 | 0.480 | 0.665 |
| LIPQOL_5 | 0.913 | 0.033 | 27.525 | < .001 | 0.848 | 0.978 |
| LIPQOL_3c | 0.402 | 0.043 | 9.304 | < .001 | 0.317 | 0.487 |
| LIPQOL_3d | 0.502 | 0.047 | 10.643 | < .001 | 0.410 | 0.595 |
| LIPQOL_3e | 0.332 | 0.038 | 8.826 | < .001 | 0.258 | 0.405 |
| LIPQOL_3f | 0.276 | 0.032 | 8.752 | < .001 | 0.214 | 0.338 |
| LIPQOL_4 | 0.267 | 0.041 | 6.518 | < .001 | 0.187 | 0.347 |
| LIPQOL_6 | 0.623 | 0.052 | 11.904 | < .001 | 0.521 | 0.726 |
| LIPQOL_7 | 0.374 | 0.050 | 7.523 | < .001 | 0.276 | 0.471 |
| LIPQOL_8 | 0.731 | 0.049 | 14.886 | < .001 | 0.634 | 0.827 |
| LIPQOL_9 | 0.316 | 0.038 | 8.384 | < .001 | 0.242 | 0.390 |
| LIPQOL_10 | 0.220 | 0.037 | 5.928 | < .001 | 0.147 | 0.293 |
| LIPQOL_11 | 0.459 | 0.050 | 9.278 | < .001 | 0.362 | 0.556 |
| LIPQOL_12 | 0.395 | 0.046 | 8.633 | < .001 | 0.305 | 0.485 |
| LIPQOL_13 | 0.231 | 0.039 | 5.879 | < .001 | 0.154 | 0.308 |
| LIPQOL_14 | 0.266 | 0.037 | 7.242 | < .001 | 0.194 | 0.338 |
| LIPQOL_15 | 0.614 | 0.051 | 12.006 | < .001 | 0.513 | 0.714 |
| LIPQOL_16 | 0.526 | 0.046 | 11.364 | < .001 | 0.435 | 0.617 |
| LIPQOL_17 | 0.424 | 0.044 | 9.542 | < .001 | 0.337 | 0.511 |
| LIPQOL_18 | 0.401 | 0.046 | 8.683 | < .001 | 0.310 | 0.491 |
| LIPQOL_19 | 0.314 | 0.052 | 6.087 | < .001 | 0.213 | 0.415 |
| LIPQOL_20 | 0.633 | 0.055 | 11.511 | < .001 | 0.525 | 0.741 |
| LIPQOL_21 | 0.456 | 0.047 | 9.606 | < .001 | 0.363 | 0.549 |
| LIPQOL_22 | 0.299 | 0.047 | 6.372 | < .001 | 0.207 | 0.391 |
| LIPQOL_23 | 0.228 | 0.036 | 6.326 | < .001 | 0.158 | 0.299 |
| LIPQOL_24 | 0.351 | 0.045 | 7.838 | < .001 | 0.263 | 0.439 |
| LIPQOL_25 | 0.404 | 0.044 | 9.130 | < .001 | 0.317 | 0.490 |

Residual covariances

95% Confidence Interval

|  |  |  | Std. estimate | Std. Error | z-value | p | Lower | Upper |
| --- | --- | --- | --- | --- | --- | --- | --- | --- |
| LIPQOL_5 | ↔ | LIPQOL_8 | 0.572 | 0.050 | 11.40 | < .001 | 0.474 | 0.671 |
| LIPQOL_17 | ↔ | LIPQOL_18 | 0.547 | 0.055 | 10.04 | < .001 | 0.440 | 0.654 |

Thresholds

95% Confidence Interval

| Indicator | Threshold | Estimate | Std. Error | z-value | p | Lower | Upper |
| --- | --- | --- | --- | --- | --- | --- | --- |
| LIPQOL_3a | t1 | −1.429 | 0.099 | −14.496 | 0.000 | −1.622 | −1.236 |
|  | t2 | −0.018 | 0.067 | −0.266 | 0.790 | −0.149 | 0.113 |
|  | t3 | 0.954 | 0.079 | 12.061 | 0.000 | 0.799 | 1.109 |
| LIPQOL_3b | t1 | −0.556 | 0.071 | −7.871 | 3.553×10^−15^ | −0.695 | −0.418 |
|  | t2 | 0.668 | 0.073 | 9.210 | 0.000 | 0.526 | 0.810 |
|  | t3 | 1.491 | 0.102 | 14.589 | 0.000 | 1.291 | 1.691 |
| LIPQOL_5 | t1 | −1.470 | 0.101 | −14.563 | 0.000 | −1.668 | −1.272 |
|  | t2 | −0.046 | 0.067 | −0.691 | 0.490 | −0.177 | 0.085 |
|  | t3 | 1.048 | 0.082 | 12.780 | 0.000 | 0.887 | 1.209 |
| LIPQOL_3c | t1 | −1.635 | 0.112 | −14.611 | 0.000 | −1.855 | −1.416 |
|  | t2 | −0.168 | 0.067 | −2.497 | 0.013 | −0.299 | −0.036 |
|  | t3 | 0.838 | 0.076 | 11.013 | 0.000 | 0.689 | 0.987 |
| LIPQOL_3d | t1 | −0.779 | 0.075 | −10.421 | 0.000 | −0.925 | −0.632 |
|  | t2 | 0.507 | 0.070 | 7.246 | 4.301×10^−13^ | 0.370 | 0.645 |
|  | t3 | 1.449 | 0.100 | 14.531 | 0.000 | 1.254 | 1.645 |
| LIPQOL_3e | t1 | −1.180 | 0.087 | −13.598 | 0.000 | −1.350 | −1.010 |
|  | t2 | 0.039 | 0.067 | 0.585 | 0.559 | −0.092 | 0.170 |
|  | t3 | 0.900 | 0.078 | 11.591 | 0.000 | 0.748 | 1.052 |
| LIPQOL_3f | t1 | −1.303 | 0.092 | −14.146 | 0.000 | −1.483 | −1.122 |
|  | t2 | 0.197 | 0.067 | 2.921 | 0.003 | 0.065 | 0.328 |
|  | t3 | 1.166 | 0.086 | 13.521 | 0.000 | 0.997 | 1.335 |
| LIPQOL_4 | t1 | −1.559 | 0.107 | −14.633 | 0.000 | −1.768 | −1.350 |
|  | t2 | −0.211 | 0.067 | −3.134 | 0.002 | −0.343 | −0.079 |
|  | t3 | 0.779 | 0.075 | 10.421 | 0.000 | 0.632 | 0.925 |
| LIPQOL_6 | t1 | −1.470 | 0.101 | −14.563 | 0.000 | −1.668 | −1.272 |
|  | t2 | −0.321 | 0.068 | −4.722 | 2.334×10^−6^ | −0.455 | −0.188 |
|  | t3 | 0.808 | 0.075 | 10.718 | 0.000 | 0.660 | 0.955 |
| LIPQOL_7 | t1 | −2.532 | 0.248 | −10.229 | 0.000 | −3.018 | −2.047 |
|  | t2 | −1.086 | 0.083 | −13.037 | 0.000 | −1.249 | −0.922 |
|  | t3 | 0.146 | 0.067 | 2.178 | 0.029 | 0.015 | 0.278 |
| LIPQOL_8 | t1 | −0.828 | 0.076 | −10.915 | 0.000 | −0.976 | −0.679 |
|  | t2 | 0.314 | 0.068 | 4.616 | 3.904×10^−6^ | 0.181 | 0.447 |
|  | t3 | 1.125 | 0.085 | 13.284 | 0.000 | 0.959 | 1.291 |
| LIPQOL_9 | t1 | −1.864 | 0.132 | −14.133 | 0.000 | −2.122 | −1.605 |
|  | t2 | −0.759 | 0.074 | −10.222 | 0.000 | −0.905 | −0.614 |
|  | t3 | 0.146 | 0.067 | 2.178 | 0.029 | 0.015 | 0.278 |
| LIPQOL_10 | t1 | −1.789 | 0.125 | −14.355 | 0.000 | −2.033 | −1.545 |
|  | t2 | −0.607 | 0.071 | −8.492 | 0.000 | −0.747 | −0.467 |
|  | t3 | 0.307 | 0.068 | 4.511 | 6.462×10^−6^ | 0.173 | 0.440 |
| LIPQOL_11 | t1 | −1.559 | 0.107 | −14.633 | 0.000 | −1.768 | −1.350 |
|  | t2 | −0.641 | 0.072 | −8.903 | 0.000 | −0.783 | −0.500 |
|  | t3 | 0.153 | 0.067 | 2.285 | 0.022 | 0.022 | 0.285 |
| LIPQOL_12 | t1 | −0.668 | 0.073 | −9.210 | 0.000 | −0.810 | −0.526 |
|  | t2 | 0.467 | 0.070 | 6.723 | 1.783×10^−11^ | 0.331 | 0.604 |
|  | t3 | 1.125 | 0.085 | 13.284 | 0.000 | 0.959 | 1.291 |
| LIPQOL_13 | t1 | −0.879 | 0.077 | −11.400 | 0.000 | −1.030 | −0.728 |
|  | t2 | 0.507 | 0.070 | 7.246 | 4.301×10^−13^ | 0.370 | 0.645 |
|  | t3 | 1.449 | 0.100 | 14.531 | 0.000 | 1.254 | 1.645 |
| LIPQOL_14 | t1 | −2.387 | 0.212 | −11.276 | 0.000 | −2.802 | −1.972 |
|  | t2 | −0.524 | 0.070 | −7.454 | 9.037×10^−14^ | −0.661 | −0.386 |
|  | t3 | 0.650 | 0.072 | 9.006 | 0.000 | 0.509 | 0.792 |
| LIPQOL_15 | t1 | −2.057 | 0.155 | −13.308 | 0.000 | −2.360 | −1.754 |
|  | t2 | −0.668 | 0.073 | −9.210 | 0.000 | −0.810 | −0.526 |
|  | t3 | 0.344 | 0.068 | 5.039 | 4.678×10^−7^ | 0.210 | 0.478 |
| LIPQOL_16 | t1 | −2.120 | 0.163 | −12.972 | 0.000 | −2.440 | −1.800 |
|  | t2 | −0.075 | 0.067 | −1.116 | 0.264 | −0.206 | 0.056 |
|  | t3 | 1.048 | 0.082 | 12.780 | 0.000 | 0.887 | 1.209 |
| LIPQOL_17 | t1 | −2.279 | 0.190 | −12.005 | 0.000 | −2.651 | −1.907 |
|  | t2 | −0.598 | 0.071 | −8.388 | 0.000 | −0.738 | −0.459 |
|  | t3 | 0.444 | 0.069 | 6.408 | 1.473×10^−10^ | 0.308 | 0.579 |
| LIPQOL_18 | t1 | −2.387 | 0.212 | −11.276 | 0.000 | −2.802 | −1.972 |
|  | t2 | −0.633 | 0.072 | −8.801 | 0.000 | −0.774 | −0.492 |
|  | t3 | 0.590 | 0.071 | 8.285 | 2.220×10^−16^ | 0.450 | 0.729 |
| LIPQOL_19 | t1 | −2.057 | 0.155 | −13.308 | 0.000 | −2.360 | −1.754 |
|  | t2 | −0.650 | 0.072 | −9.006 | 0.000 | −0.792 | −0.509 |
|  | t3 | 0.336 | 0.068 | 4.933 | 8.078×10^−7^ | 0.203 | 0.470 |
| LIPQOL_20 | t1 | −1.060 | 0.082 | −12.867 | 0.000 | −1.222 | −0.899 |
|  | t2 | 0.168 | 0.067 | 2.497 | 0.013 | 0.036 | 0.299 |
|  | t3 | 1.012 | 0.081 | 12.516 | 0.000 | 0.853 | 1.170 |
| LIPQOL_21 | t1 | −0.818 | 0.076 | −10.817 | 0.000 | −0.966 | −0.669 |
|  | t2 | 0.299 | 0.068 | 4.405 | 1.058×10^−5^ | 0.166 | 0.432 |
|  | t3 | 1.303 | 0.092 | 14.146 | 0.000 | 1.122 | 1.483 |
| LIPQOL_22 | t1 | −1.036 | 0.082 | −12.693 | 0.000 | −1.196 | −0.876 |
|  | t2 | 0.132 | 0.067 | 1.966 | 0.049 | 4.043×10^−4^ | 0.263 |
|  | t3 | 0.977 | 0.080 | 12.245 | 0.000 | 0.821 | 1.133 |
| LIPQOL_23 | t1 | −1.036 | 0.082 | −12.693 | 0.000 | −1.196 | −0.876 |
|  | t2 | 0.204 | 0.067 | 3.028 | 0.002 | 0.072 | 0.336 |
|  | t3 | 0.900 | 0.078 | 11.591 | 0.000 | 0.748 | 1.052 |
| LIPQOL_24 | t1 | −0.808 | 0.075 | −10.718 | 0.000 | −0.955 | −0.660 |
|  | t2 | 0.548 | 0.071 | 7.767 | 7.994×10^−15^ | 0.410 | 0.687 |
|  | t3 | 1.354 | 0.095 | 14.315 | 0.000 | 1.169 | 1.540 |
| LIPQOL_25 | t1 | −0.722 | 0.074 | −9.820 | 0.000 | −0.866 | −0.578 |
|  | t2 | 0.499 | 0.070 | 7.141 | 9.244×10^−13^ | 0.362 | 0.636 |
|  | t3 | 1.337 | 0.094 | 14.261 | 0.000 | 1.153 | 1.520 |

Reliability

|  | Coefficient ω | Coefficient α |
| --- | --- | --- |
| Funksjon | 0.904 | 0.861 |
| Kroppsbilde | 0.902 | 0.869 |
| Symptomer | 0.850 | 0.843 |
| Følelser | 0.906 | 0.859 |
| total | 0.964 | 0.930 |

Modification Indices

Cross-loadings

|  |  |  | Mod. Ind. | EPC |
| --- | --- | --- | --- | --- |
| Funksjon | → | LIPQOL_19 | 55.719 | −1.063 |
| Kroppsbilde | → | LIPQOL_5 | 51.372 | −0.556 |
| Kroppsbilde | → | LIPQOL_4 | 48.035 | −0.462 |
| Kroppsbilde | → | LIPQOL_19 | 37.629 | −0.467 |
| Funksjon | → | LIPQOL_16 | 36.543 | 0.841 |
| Symptomer | → | LIPQOL_20 | 35.321 | −0.256 |
| Funksjon | → | LIPQOL_20 | 31.342 | −0.260 |
| Følelser | → | LIPQOL_9 | 31.199 | 0.425 |
| Funksjon | → | LIPQOL_9 | 30.539 | 0.281 |
| Funksjon | → | LIPQOL_8 | 29.301 | 0.277 |
| Følelser | → | LIPQOL_19 | 27.057 | −0.387 |
| Symptomer | → | LIPQOL_8 | 26.826 | 0.269 |
| Følelser | → | LIPQOL_4 | 25.077 | −0.345 |
| Følelser | → | LIPQOL_16 | 22.585 | 0.364 |
| Symptomer | → | LIPQOL_25 | 22.300 | 0.204 |
| Funksjon | → | LIPQOL_25 | 19.532 | 0.201 |
| Symptomer | → | LIPQOL_9 | 19.013 | 0.223 |
| Funksjon | → | LIPQOL_14 | 14.577 | −0.531 |
| Kroppsbilde | → | LIPQOL_3f | 14.049 | 0.267 |
| Funksjon | → | LIPQOL_13 | 12.600 | −0.173 |
| Funksjon | → | LIPQOL_12 | 11.783 | −0.166 |
| Følelser | → | LIPQOL_12 | 11.368 | −0.242 |
| Symptomer | → | LIPQOL_13 | 10.912 | −0.165 |
| Følelser | → | LIPQOL_3a | 10.887 | 0.244 |
| Kroppsbilde | → | LIPQOL_16 | 8.343 | 0.230 |
| Funksjon | → | LIPQOL_10 | 8.038 | 0.142 |
| Symptomer | → | LIPQOL_10 | 7.934 | 0.144 |
| Symptomer | → | LIPQOL_24 | 7.876 | 0.120 |
| Symptomer | → | LIPQOL_12 | 7.718 | −0.139 |
| Symptomer | → | LIPQOL_22 | 7.535 | −0.115 |
| Funksjon | → | LIPQOL_6 | 7.470 | −0.136 |
| Følelser | → | LIPQOL_13 | 7.459 | −0.199 |
| Kroppsbilde | → | LIPQOL_20 | 7.147 | −0.183 |
| Funksjon | → | LIPQOL_24 | 6.974 | 0.119 |
| Funksjon | → | LIPQOL_17 | 6.709 | 0.359 |
| Kroppsbilde | → | LIPQOL_24 | 6.700 | 0.174 |
| Funksjon | → | LIPQOL_15 | 6.474 | 0.362 |
| Følelser | → | LIPQOL_15 | 6.435 | 0.194 |
| Følelser | → | LIPQOL_8 | 6.390 | 0.189 |
| Følelser | → | LIPQOL_14 | 6.221 | −0.185 |
| Kroppsbilde | → | LIPQOL_25 | 5.862 | 0.157 |
| Symptomer | → | LIPQOL_3a | 5.852 | −0.281 |
| Symptomer | → | LIPQOL_4 | 5.807 | −0.279 |
| Symptomer | → | LIPQOL_5 | 5.799 | −0.364 |
| Funksjon | → | LIPQOL_22 | 5.425 | −0.103 |
| Følelser | → | LIPQOL_5 | 5.254 | −0.173 |
| Symptomer | → | LIPQOL_3f | 5.210 | 0.274 |
| Symptomer | → | LIPQOL_7 | 4.958 | −0.113 |
| Kroppsbilde | → | LIPQOL_3e | 4.711 | 0.155 |
| Funksjon | → | LIPQOL_18 | 4.556 | 0.301 |
| Symptomer | → | LIPQOL_6 | 4.458 | −0.107 |

| Residual covariances | | | | |
| --- | --- | --- | --- | --- |
|  |  |  | Mod. Ind. | EPC |
| LIPQOL_16 | ↔ | LIPQOL_18 | 47.867 | −0.261 |
| LIPQOL_9 | ↔ | LIPQOL_10 | 33.762 | −0.181 |
| LIPQOL_24 | ↔ | LIPQOL_25 | 31.889 | −0.197 |
| LIPQOL_16 | ↔ | LIPQOL_17 | 20.492 | −0.183 |
| LIPQOL_14 | ↔ | LIPQOL_19 | 18.409 | 0.204 |
| LIPQOL_5 | ↔ | LIPQOL_7 | 16.049 | −0.227 |
| LIPQOL_18 | ↔ | LIPQOL_19 | 15.593 | 0.210 |
| LIPQOL_12 | ↔ | LIPQOL_13 | 14.916 | −0.138 |
| LIPQOL_4 | ↔ | LIPQOL_19 | 13.849 | −0.129 |
| LIPQOL_7 | ↔ | LIPQOL_13 | 13.485 | 0.190 |
| LIPQOL_14 | ↔ | LIPQOL_20 | 11.881 | −0.181 |
| LIPQOL_5 | ↔ | LIPQOL_6 | 11.009 | −0.187 |
| LIPQOL_20 | ↔ | LIPQOL_23 | 10.929 | 0.175 |
| LIPQOL_8 | ↔ | LIPQOL_9 | 10.783 | −0.159 |
| LIPQOL_7 | ↔ | LIPQOL_19 | 10.734 | −0.158 |
| LIPQOL_6 | ↔ | LIPQOL_7 | 10.719 | −0.146 |
| LIPQOL_23 | ↔ | LIPQOL_25 | 10.444 | −0.116 |
| LIPQOL_7 | ↔ | LIPQOL_12 | 10.238 | 0.175 |
| LIPQOL_4 | ↔ | LIPQOL_6 | 10.033 | −0.147 |
| LIPQOL_3f | ↔ | LIPQOL_9 | 9.488 | 0.184 |
| LIPQOL_17 | ↔ | LIPQOL_19 | 9.428 | 0.153 |
| LIPQOL_3a | ↔ | LIPQOL_19 | 9.307 | −0.118 |
| LIPQOL_16 | ↔ | LIPQOL_19 | 9.049 | 0.189 |
| LIPQOL_9 | ↔ | LIPQOL_12 | 8.969 | 0.151 |
| LIPQOL_4 | ↔ | LIPQOL_7 | 8.866 | −0.132 |
| LIPQOL_9 | ↔ | LIPQOL_13 | 8.642 | 0.142 |
| LIPQOL_4 | ↔ | LIPQOL_13 | 8.600 | −0.124 |
| LIPQOL_5 | ↔ | LIPQOL_3f | 8.518 | 0.172 |
| LIPQOL_20 | ↔ | LIPQOL_24 | 8.286 | 0.154 |
| LIPQOL_10 | ↔ | LIPQOL_13 | 8.090 | 0.120 |
| LIPQOL_21 | ↔ | LIPQOL_25 | 8.070 | 0.139 |
| LIPQOL_4 | ↔ | LIPQOL_12 | 8.055 | −0.130 |
| LIPQOL_3b | ↔ | LIPQOL_6 | 7.933 | −0.147 |
| LIPQOL_6 | ↔ | LIPQOL_12 | 7.386 | 0.152 |
| LIPQOL_22 | ↔ | LIPQOL_25 | 7.235 | 0.123 |
| LIPQOL_5 | ↔ | LIPQOL_9 | 7.182 | −0.156 |
| LIPQOL_3c | ↔ | LIPQOL_4 | 6.991 | 0.112 |
| LIPQOL_10 | ↔ | LIPQOL_12 | 6.947 | 0.121 |
| LIPQOL_3e | ↔ | LIPQOL_3f | 6.467 | −0.078 |
| LIPQOL_3d | ↔ | LIPQOL_9 | 6.154 | 0.147 |
| LIPQOL_3f | ↔ | LIPQOL_10 | 6.085 | 0.142 |
| LIPQOL_3e | ↔ | LIPQOL_9 | 6.079 | 0.148 |
| LIPQOL_3e | ↔ | LIPQOL_4 | 6.070 | 0.097 |
| LIPQOL_3d | ↔ | LIPQOL_3e | 5.944 | −0.088 |
| LIPQOL_3d | ↔ | LIPQOL_19 | 5.761 | 0.131 |
| LIPQOL_15 | ↔ | LIPQOL_23 | 5.668 | 0.147 |
| LIPQOL_21 | ↔ | LIPQOL_23 | 5.630 | 0.110 |
| LIPQOL_3d | ↔ | LIPQOL_4 | 5.542 | 0.116 |
| LIPQOL_3f | ↔ | LIPQOL_8 | 5.487 | 0.135 |
| LIPQOL_17 | ↔ | LIPQOL_25 | 5.437 | 0.145 |
| LIPQOL_6 | ↔ | LIPQOL_19 | 5.413 | −0.117 |
| LIPQOL_5 | ↔ | LIPQOL_10 | 5.093 | −0.129 |
| LIPQOL_16 | ↔ | LIPQOL_23 | 4.953 | 0.140 |
| LIPQOL_9 | ↔ | LIPQOL_22 | 4.875 | 0.125 |
| LIPQOL_19 | ↔ | LIPQOL_22 | 4.854 | −0.111 |
| LIPQOL_8 | ↔ | LIPQOL_18 | 4.844 | 0.135 |
| LIPQOL_14 | ↔ | LIPQOL_22 | 4.773 | −0.110 |
| LIPQOL_8 | ↔ | LIPQOL_14 | 4.710 | 0.124 |
| LIPQOL_7 | ↔ | LIPQOL_9 | 4.656 | −0.090 |
| LIPQOL_13 | ↔ | LIPQOL_19 | 4.629 | −0.106 |
| LIPQOL_3a | ↔ | LIPQOL_10 | 4.540 | 0.121 |
| LIPQOL_7 | ↔ | LIPQOL_25 | 4.510 | 0.129 |
| LIPQOL_3d | ↔ | LIPQOL_8 | 4.475 | 0.117 |
| LIPQOL_12 | ↔ | LIPQOL_19 | 4.439 | −0.106 |
| LIPQOL_17 | ↔ | LIPQOL_20 | 4.424 | −0.114 |
| LIPQOL_21 | ↔ | LIPQOL_22 | 4.357 | −0.083 |
| LIPQOL_8 | ↔ | LIPQOL_10 | 4.199 | −0.103 |
| LIPQOL_3a | ↔ | LIPQOL_3b | 4.142 | −0.087 |
| LIPQOL_5 | ↔ | LIPQOL_11 | 4.142 | −0.125 |
| LIPQOL_19 | ↔ | LIPQOL_21 | 3.990 | −0.108 |
| LIPQOL_20 | ↔ | LIPQOL_21 | 3.920 | −0.089 |
