# Supplementary material for: Initial psychometric evaluation of the Lipedema Quality of Life questionnaire in females with lipedema in Norway
Source: Front Glob Womens Health. 2026 Oct 9;7:1917274. doi: 10.3389/fgwh.2026.1917274 (PMC13692784; doi:10.3389/fgwh.2026.1917274)
Supplement: Supplementary file 6 [file Table6.docx]

| Tysk | Norsk |
| --- | --- |
| Gar nicht | Ikke i det hele tatt |
| etwas | Litt |
| Recht stark | Ganske mye |
| Sehr stark | Svært mye |
| n. z. nicht zutreffend | Ikke aktuelt |
| 1. Wie stark empfinden Sie die Umfangsvermehrung der Beine? | 1. Hvor mye påvirkes du av økt beinomkrets? |
| 2. Wie stark empfinden Sie die Schmerzen der Beine? | 2. Hvor mye påvirkes du av smertene i beina? |
| 3. Wie stark beeinträchtigen die dicken, schmerzenden Beine und ihre Behandlung Sie bei folgenden Alltagsaktivitäten? | 3. Hvor mye begrenser de hovne og smertefulle benene og behandlingen deg i følgende daglige aktiviteter? |
| 3a. Gehen | 3a. Gåing |
| 3b. Vornüberbeugen, z.B. beim Schuhe anziehen oder der Fußpflege | b) Bøye deg fremover, f.eks. ved påkledning av sko eller fotpleie |
| 3c. Stehen | 3c. Ståing |
| 3d. Im Sitzen | 3d. Sitting |
| 3e. Im Beruf | 3e. På jobb |
| 3f. Bei der Hausarbeit | 3f. Husarbeid |
| 4. Wie stark sind Ihre Freizeitaktivitäten / Ihr Sozialleben dadurch beeinträchtigt?   Bitte nennen Sie Beispiele: | 4. Hvor mye påvirkes fritidsaktivitetene dine/ditt sosiale liv av dette?  Vennligst gi eksempler: |
| 5. Wie sehr beeinträchtigt Sie die Kompressionsbekleidung? | 5. Hvor plagsomt opplever du det er å bruke kompresjonstøy? |
| 6. Wie schwierig ist es, Kleidung zu finden, die Sie gerne tragen? | 6. Hvor mye vansker har du med å finne klær du liker å bruke? |
| 7. Wie sehr, denken Sie, beeinflusst das Lipödem Ihr Aussehen? | 7. Hvor mye mener du at lipødem påvirker utseendet ditt? |
| 8. Wie sehr, denken Sie, beeinflusst die Kompressionsbekleidung Ihr Selbstbewusstsein? | 8. Hvor mye mener du at bruk av kompresjonstøy påvirker selvtilliten din negativt? |
| 9. Beeinflussen Ihre dicken Beine Ihr Selbstbild? | 9. Hvor mye påvirker de hovne bena ditt selvbilde? |
| 10. Beeinflusst das Lipödem Ihr Selbstwertgefühl? | 10. Hvor mye påvirker lipødem selvtilliten din? |
| 11. Wie sehr, denken Sie, beeinflusst das Lipödem, ob Sie sich öffentlich zeigen (bspw. Schwimmbad, Tanzen)? | 11. Hvor mye mener du at lipødem påvirker om du viser deg i offentlige sammenhenger (f.eks. i svømmehallen, dans etc.) |
| 12. Beeinflusst das Lipödem Ihre Partnerschaft? | 12. Påvirker lipødem ditt parforhold? |
| 13. Beeinflusst das Lipödem Ihre Beziehungen zu anderen Menschen? | 13. Påvirker lipødem dine relasjoner til andre mennesker? |
| 14. Wie stark empfinden Sie die Schmerzen durch das Lipödem? | 14. Hvor mye smerter opplever du på grunn av lipødem? |
| 15. Wie stark ist der Schmerz bei Druck und Berührung? | 15. Hvor mye smerter opplever du ved trykk og berøring? |
| 16. Schwellen Ihre Beine im Tagesverlauf an? | 16. Hovner bena dine opp i løpet av dagen? |
| 17. Haben Sie ein Spannungsgefühl in den Beinen? | 17. Har du følelse av trykk/press i bena? |
| 18. Werden Ihre Beine im Tagesverlauf schwerer? | 18. Blir beina tyngre utover dagen? |
| 19. Beeinträchtigt das Lipödem Ihre körperliche Leistungsfähigkeit? | 19. Reduserer lipødem din fysiske prestasjonsevne? |
| Hatten Sie in der vergangenen Woche… | Har du i løpet av den siste uken hatt.... |
| 20. Probleme mit Schlaf? | 20. Søvnproblemer? |
| 21. Konzentrationsschwierigkeiten, z.B. beim Lesen? | 21. Konsentrasjonsvansker, f.eks. ved lesing? |
| 22. Gefühle der Anspannung? | 22. Følelse av anspenthet? |
| 23. Sorgen? | 23. Bekymringer? |
| 24. Erhöhte Reizbarkeit? | 24. Økt irritabilitet? |
| 25. Niedergeschlagenheit? | 25. Nedstemthet? |
| 26. Wie würden Sie Ihre Lebensqualität derzeit insgesamt bewerten? | 26. Hvordan vil du vurdere din nåværende livskvalitet totalt sett? |
| Bitte vergeben Sie einen Punktwert auf der folgenden Skala: | Vennligst kryss av på følgende skala: |
| extrem schlecht | Svært dårlig |
| hervorragend | Svært bra |
| Vielen Dank für Ihre Teilnahme! | Tusen takk for din deltakelse! |
